# Robust hematopoietic specification requires the ubiquitous Sp1 and Sp3 transcription factors

**DOI:** 10.1186/s13072-019-0282-9

**Published:** 2019-06-04

**Authors:** Jane Gilmour, Leigh O’Connor, Christopher P. Middleton, Peter Keane, Nynke Gillemans, Jean-Baptiste Cazier, Sjaak Philipsen, Constanze Bonifer

**Affiliations:** 1 Institute of Cancer and Genomic Sciences, University of Birmingham, Birmingham, UK; 2 Centre for Computational Biology, University of Birmingham, Birmingham, UK; 3 Department of Cell Biology, Erasmus MC, Rotterdam, The Netherlands

**Keywords:** Blood cell development, ATAC-seq, Single-cell RNA-seq, Embryonic development, Differentiation trajectories, Transcription factor cooperation

## Abstract

**Background:**

Both tissue-specific and ubiquitously expressed transcription factors, such as Sp-family members, are required for correct development. However, the molecular details of how ubiquitous factors are involved in programming tissue-specific chromatin and thus participate in developmental processes are still unclear. We previously showed that embryonic stem cells lacking Sp1 DNA-binding activity (Sp1^ΔDBD/ΔDBD^ cells) are able to differentiate into early blood progenitors despite the inability of Sp1 to bind chromatin without its DNA-binding domain. However, gene expression during differentiation becomes progressively deregulated, and terminal differentiation is severely compromised.

**Results:**

Here, we studied the cooperation of Sp1 with its closest paralogue Sp3 in hematopoietic development and demonstrate that Sp1 and Sp3 binding sites largely overlap. The complete absence of either Sp1 or Sp3 or the presence of the Sp1 DNA-binding mutant has only a minor effect on the pattern of distal accessible chromatin sites and their transcription factor binding motif content, suggesting that these mutations do not affect tissue-specific chromatin programming. Sp3 cooperates with Sp1^ΔDBD/ΔDBD^ to enable hematopoiesis, but is unable to do so in the complete absence of Sp1. Using single-cell gene expression analysis, we show that the lack of Sp1 DNA binding leads to a distortion of cell fate decision timing, indicating that stable chromatin binding of Sp1 is required to maintain robust differentiation trajectories.

**Conclusions:**

Our findings highlight the essential contribution of ubiquitous factors such as Sp1 to blood cell development. In contrast to tissue-specific transcription factors which are required to direct specific cell fates, loss of Sp1 leads to a widespread deregulation in timing and coordination of differentiation trajectories during hematopoietic specification.

**Electronic supplementary material:**

The online version of this article (10.1186/s13072-019-0282-9) contains supplementary material, which is available to authorized users.

## Background

The interaction of transcription factors (TFs) with chromatin drives the cooperation of distal elements with promoters and directs cell fate choices in a developmental context. Tissue-specific gene activity is mostly driven by distal elements such as enhancers bound by tissue-specific factors, and their activity strongly correlates with gene expression patterns. In contrast, promoter elements, in particular CG island promoters, are enriched for motifs of ubiquitously expressed factors such as NFY or Sp1 and do not show such strict correlation [1]. Moreover, distal elements can be activated at the chromatin level prior to the onset of gene expression, and subsequently drive tissue-specific gene activity by interaction with the generic factors bound to promoters [2]. However, the precise role of ubiquitously expressed TFs in the regulation of tissue-specific gene expression is not well understood.

Sp1 was the first identified member of a large family of zinc finger transcription factors recognising the GC and GT box DNA elements with binding sites in vivo being enriched for CG island promoters [3–6]. Both Sp1 and the closely related family member Sp3 are ubiquitously expressed and recognise the same DNA-binding motifs. Sp1 DNA binding is indispensable for normal mammalian embryo development, since mice expressing a truncated version of Sp1 lacking the DNA-binding domain (DBD) (Sp1^ΔDBD/ΔDBD^) demonstrate multiple heterogeneous phenotypic abnormalities and die in utero [7]. It was not possible to identify a specific role of Sp1 in a specific pathway by conditional deletion, as no defects were observed when the gene was deleted at later stages of development [3] (unpublished results), indicating that the developmental defects in Sp1^ΔDBD/ΔDBD^ mice were cumulative. Sp3^−/−^ mice with a complete gene knockout survive until birth but die shortly after due to respiratory failure [8]. Additional studies in Sp3-null mice provided evidence of defects in hematopoiesis and a requirement for Sp3 in normal cardiac development [9, 10]. Sp1^+/ΔDBD^/Sp3^+/−^ compound heterozygous mice also showed embryonic lethality but survived until later in development than Sp1^ΔDBD/ΔDBD^ mice [11]. Therefore, while there may be some functional redundancy, tightly regulated levels of both factors are required throughout normal embryogenesis. However, the extent of the cooperativity of these two proteins at the genomic level has not been fully resolved. In addition, it is unknown whether in the absence of the DBD, the Sp1 protein is capable of stable interaction with the genome.

Sp3 has been described as a repressor of Sp1 transactivation and was proposed to exert this effect by competing for the Sp binding motif, since Sp3 lacking a DNA-binding domain could not inhibit Sp1 activity [12]. However, Sp3 has been shown to have transactivation potential, and several studies have demonstrated synergistic activation of target genes by Sp1 and Sp3 [5, 13, 14]. Volkel et al. [15] found that binding of Sp1 and Sp3 in mouse embryonic fibroblasts (MEFs) largely overlapped and binding was predominantly at promoter sites with conventional GC box motifs. Differences in the ability of Sp1 and Sp3 to bind to multiple GC boxes may contribute to their capacity to activate or repress at different binding sites [16, 17]. The issue of co-localisation is still not clear as He et al. [18] demonstrated localisation of the two proteins to different nuclear compartments in MCF7 cells.

To be able to conduct genome-wide binding studies and to bypass the problems with embryonic lethality in mice, we used in vitro differentiation of mouse embryonic stem cells (ESC) into blood cells as a tractable model to shed light on the role of Sp1 in the control of developmental gene expression. We previously showed that Sp1^ΔDBD/ΔDBD^ cells are unable to terminally differentiate and that this phenotype was associated with a progressive deregulation in gene expression [3]. In the present study, we aimed to investigate (1) the phenotype of ESC with a complete knockout of Sp1, (2) the ability of Sp3 to compensate for the absence/dysfunction of Sp1 and (3) whether the progressive deregulation of gene expression in differentiating Sp1^ΔDBD/ΔDBD^ cells was due to heterogeneity of gene expression within cells or a failure of executing cell fate decisions. Our results demonstrate that Sp1 and Sp3 binding sites strongly overlap and that the Sp1^ΔDBD/ΔDBD^ protein partially retains Sp1 function. While Sp3 can compensate for Sp1 binding at some target genes, it cannot support hematopoietic specification alone, and Sp3-null ESC also have a defect in myeloid differentiation. Most importantly, we show by single-cell RNA sequencing that the differentiation trajectory leading to hematopoietic differentiation is severely disturbed in Sp1^ΔDBD/ΔDBD^ cells. Cells execute the order of cell fate decisions correctly, but not as a cohort and with delayed kinetics, indicating that Sp1 is required for executing a robust differentiation program.

## Results

### The complete knockout of Sp1 is incompatible with hematopoietic specification but does not lead to widespread changes in chromatin accessibility

**Fig. 1:**
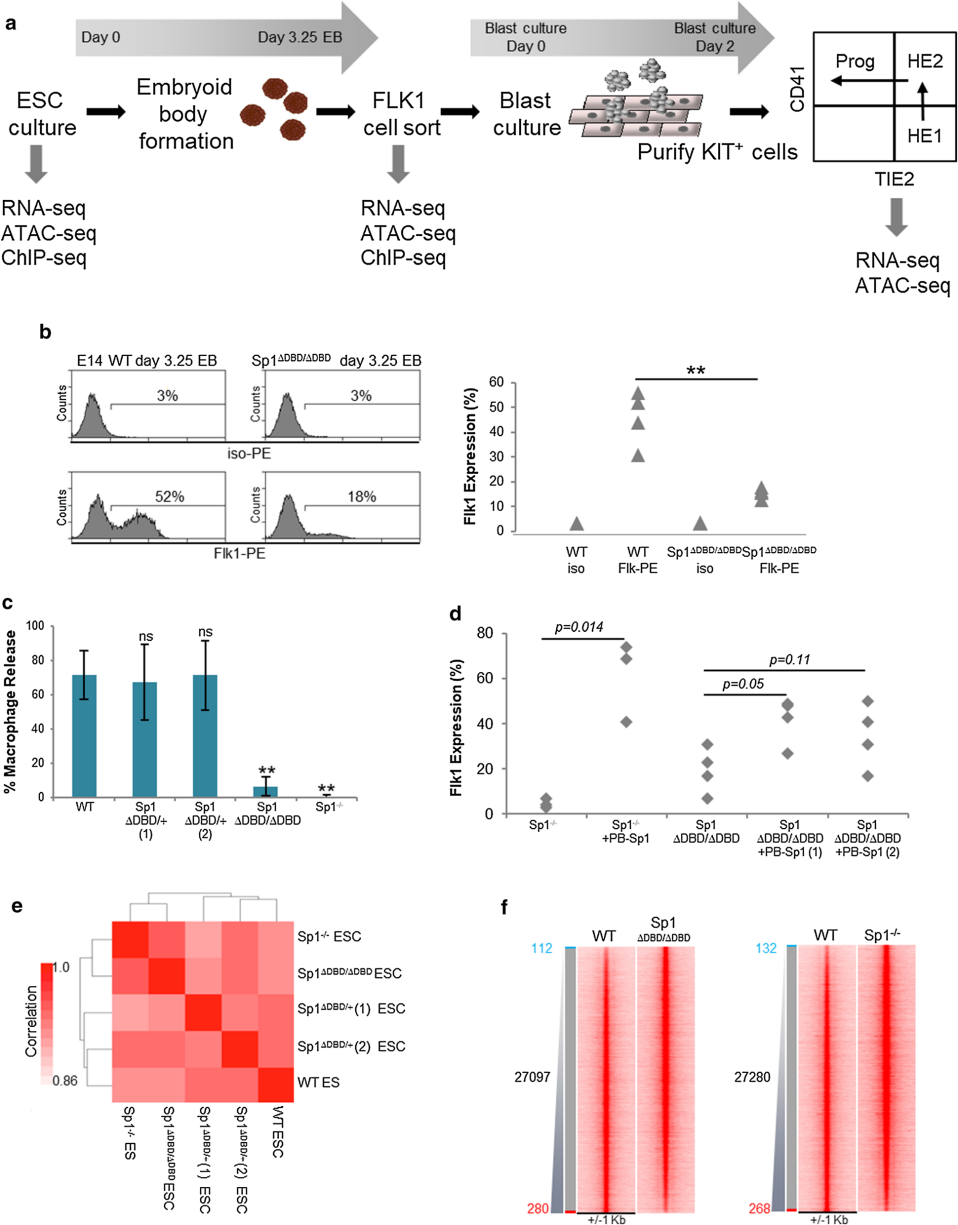
The complete absence or the truncation  of Sp1 do not cause widespread changes in chromatin accessibility. **a** Schematic representing in vitro differentiation of ESC. **b** FACS analysis of Flk1 expression in E14 WT and E14 Sp1^ΔDBD/ΔDBD^ cells derived from embryoid bodies (EB) at day 3.25 of in vitro differentiation. Left panel: representative FACS profiles for Flk1-PE staining, right panel: graph showing Flk1 expression and isotype controls for E14 WT and Sp1^ΔDBD/ΔDBD^ cells (*n* = 4, **indicates *p* < 0.001). **c** Graph showing the percentage of EB releasing macrophages in a macrophage release assay (*n* = 3, * indicates *p* < 0.05). **d** Rescue of Flk1 expression levels by re-expression of wild-type Sp1 in Sp1^−/−^ and Sp1^ΔDBD/ΔDBD^ cells (*n* = 3 for Sp1^−/−^ and *n* = 4 for Sp1^ΔDBD/ΔDBD^, *p* values are indicated on the graph). **e** Pearson correlation plot of accessible chromatin regions in ESC as determined by ATAC-seq, in WT cells and Sp1 mutant ESC clones. **f** Heat maps showing the fold difference in accessible chromatin sites, as determined by ATAC, between WT and Sp1^ΔDBD/ΔDBD^ E14 ESC (left panel) and WT and Sp1^−/−^ A17Lox ESC (right panel). The red box indicates WT-specific ATAC sites, while the blue box indicates ATAC sites specific to either Sp1^ΔDBD/ΔDBD^ or Sp1^−/−^ cell lines

Using an in vitro hematopoietic differentiation system [19, 20] (Fig. 1a), we previously demonstrated that murine E14 ESC lacking the DBD of Sp1 (E14 Sp1^ΔDBD/ΔDBD^) show a defect in macrophage differentiation. We show here that these cells also have a reduced capacity to generate Flk1 + hemangioblast cells (Fig. 1b) [3]. We applied CRISPR-Cas9 gene editing using guide RNAs targeting the DBD of Sp1 encoded by exons 5 and 6 to recapitulate this defect in a different ES cell type (A17 2Lox cells) [21, 22] (Additional file 1: Fig. S1a). Gene editing generated a clone (A17Lox Sp1^ΔDBD/ΔDBD^) that expressed a low level of the truncated version of Sp1 in the absence of the full-length protein (Additional file 1: Fig. S1b and S1c, clone 4). Three heterozygous clones expressing wild-type levels of Sp1 were also identified along with a clone completely lacking Sp1 protein expression (Additional file 1: Fig. S1b and S1c, clone 14). Further analysis of clone 14 revealed an out of frame mutation at the cut site in exon 5 (Additional file 1: Fig. S1a), which probably caused RNA degradation by nonsense-mediated decay, resulting in a Sp1^−/−^ phenotype.

We next evaluated the effect of CRISPR deletion in the A17Lox Sp1^ΔDBD/ΔDBD^ and A17Lox Sp1^–/−^ clones in the in vitro differentiation system and in macrophage release assays. As found with E14 Sp1^ΔDBD/ΔDBD^ cells, A17Lox Sp1^ΔDBD/ΔDBD^ cells had a significantly reduced capacity to generate Flk1 + cells from embryoid bodies (EB) while A17Lox Sp1^−/−^ cells  produced even lower levels of Flk1 + cells  (Additional file 1: Fig. S1d). Heterozygous clones generated wild-type numbers of macrophage-releasing EBs, whereas EBs from A17Lox Sp1^ΔDBD/ΔDBD^ and A17Lox Sp1^−/−^ clones had significantly lower capacity to generate macrophages with  the severest phenotype exhibited by the cells carrying a complete knockout of Sp1 (clone 14, Fig. 1c). To verify that the reduced Flk1 expression and macrophage production were a direct result of Sp1 disruption and not a result of off-target CRISPR effects, we rescued the phenotype by expressing human wild-type Sp1 (Additional file 1: Fig. S1e) and restored increased levels of Flk1 + expression as detected by FACS analysis (Fig. 1d). These data demonstrate that a complete lack of Sp1 is incompatible with the differentiation of ESC and that the truncated version of Sp1 lacking DNA binding is a hypomorph that partly retains normal Sp1 function.

To examine whether the decreased differentiation potential in the Sp1-disrupted clones was a result of changes in chromatin accessibility caused by a lack of Sp1 binding, we employed the genome-wide Assay for Transposase-Accessible Chromatin using sequencing (ATAC-seq) [23]. We found a high degree of correlation in DNA accessibility between the A17Lox WT, heterozygous and Sp1-disrupted clones (Fig. 1e). Only around 400 accessible chromatin sites were either lost or gained between the A17Lox WT cells and either A17Lox Sp1^ΔDBD/ΔDBD^ or A17Lox Sp1^−/−^ clones suggesting that lack of Sp1 does not result in widespread changes in chromatin accessibility in ESC (Fig. 1f). In addition, we confirmed similarity in hypersensitive site profiles between the A17Lox WT cells and the E14 WT cells used in the original study (Additional file 1: Fig. S1f), confirming that this phenotype was not cell clone dependent. Finally, chromatin accessibility clustered by cell type rather than by Sp1 binding capacity when we compared ESC and Flk1 + differentiation stages (Additional file 1: Fig. S1g), indicating that the developmentally regulated activation and silencing of active cis-regulatory elements which exists as accessible chromatin sites was not affected by the absence of Sp1. While there were some sites such as an intronic region of the *Ostm1* gene that displayed a loss of accessibility specifically in the Sp1-disrupted clones (Additional file 1: Fig. S1h), the vast majority of accessible sites were unaffected as observed at the *Pou5f1* locus (Additional file 1: Fig. S1i).

### Sp3 partially compensates for Sp1 in cells with disrupted Sp1 DNA binding

**Fig. 2:**
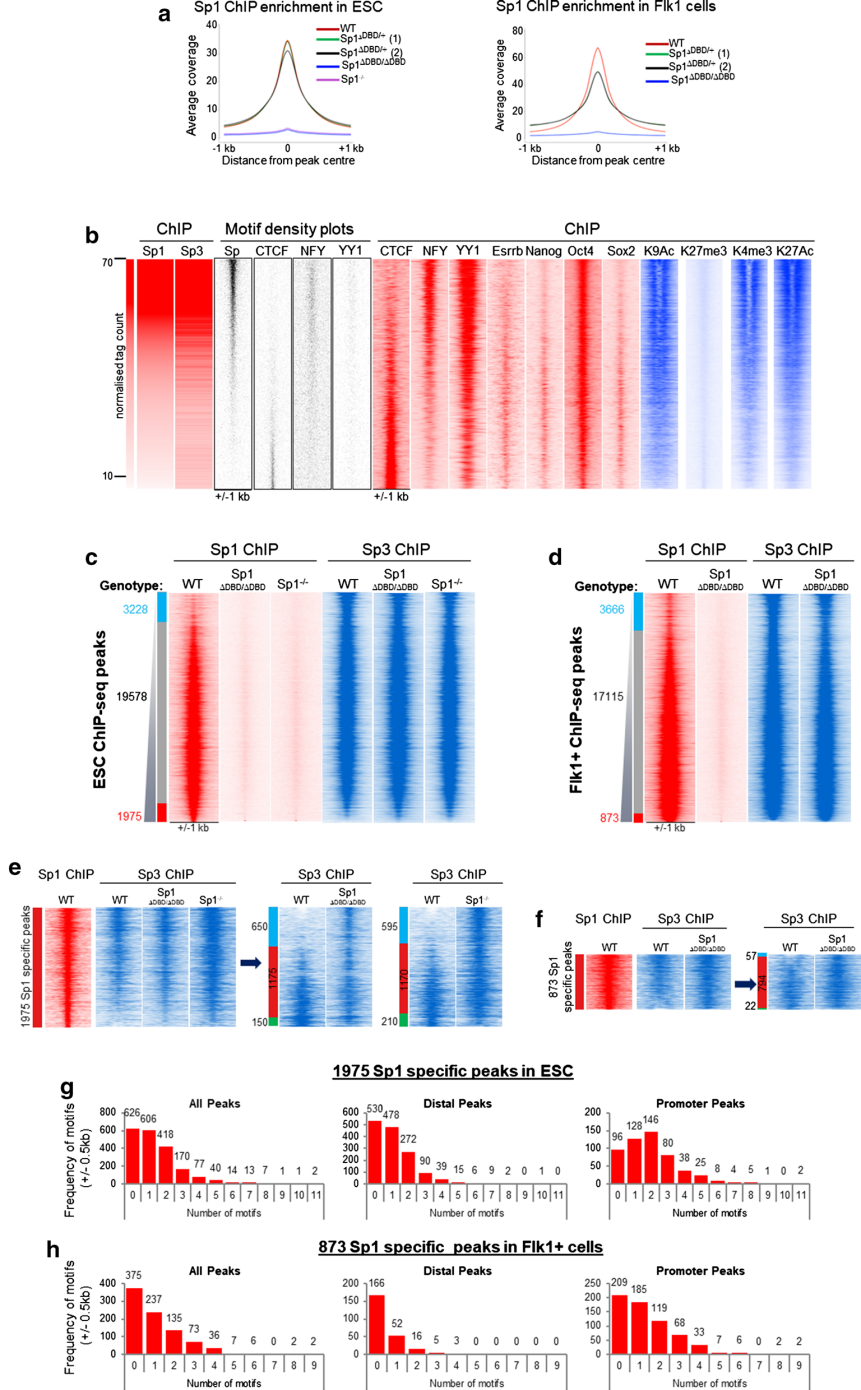
Sp3 partially compensates for the absence of Sp1 in cells with disrupted Sp1 binding. **a** Average profiles of Sp1 ChIP enrichment in ESC (left panel) and Flk1+ cells for WT and CRISPR clones. **b** Heat maps showing a ranking of normalised tag counts from Sp1 ChIP-seq in A17Lox WT ES cells. Ranked alongside are tag counts from the Sp3 ChIP-Seq in the same cells and motifs for CTCF, NFY, YY1, ESRRB, NANOG, OCT4 and SOX2. Heat maps (public datasets, see Additional file 1: Table 1) showing ChIP-seq enrichment for CTCF, NFY, YY1, ESRRB, NANOG, OCT4 and SOX2. The histone modifications H3K9Ac, H3K27me3, H3K4me3 and H3K27Ac are shown alongside. **c** Heat maps showing fold difference in ChIP-seq enrichment between Sp1 and Sp3 ChIP in E14 WT ES cells. Ranked along the same coordinates are Sp1 and Sp3 ChIP tag counts in Sp1^ΔDBD/ΔDBD^ and Sp1^−/−^  cells. Peaks were classed as specific if they showed equal to or more than twofold change in tag count difference between the Sp1 and Sp3 ChIPs. The specific and shared groups are indicated by coloured bars alongside (red indicates Sp1 specific peaks and blue indicates Sp3 specific peaks), and the number of peaks within each group is shown. **d** Heat maps showing fold difference in ChIP-seq tag count enrichment between Sp1 and Sp3 ChIP peaks in Flk1+ cells. Ranked along the same coordinates are Sp1 and Sp3 ChIP in Sp1^ΔDBD/ΔDBD^  cells. Peaks were classed as specific if they showed more than twofold change in tag count difference between the Sp1 and Sp3 ChIPs. The specific and shared groups are indicated by coloured bars alongside (red indicates Sp1 specific peaks and blue indicates Sp3 specific peaks), and the number of peaks within each group is shown. **e** Zoomed-in heat map of the 1975 Sp1 specific sites in ESC shown in Fig. 2c. Right-hand panels show the Sp3 ChIP-seq enrichment ranked according to the changes in Sp3 occupancy at these sites in Sp1^ΔDBD/ΔDBD^ and Sp1^−/−^  cells. The blue bar indicates peaks which are increased in the mutant cell lines compared to WT, and the green bar indicates peaks which are reduced at least twofold in the mutant cell lines compared to WT. **f** Zoomed-in heat map of 873 Sp1 specific sites in Flk1+ cells shown in Fig. 2d. Right-hand panel shows the Sp3 ChIP-seq enrichment ranked according to the changes in Sp3 occupancy at these sites. The blue bar indicates peaks which are increased in the mutant cell lines compared to WT, and the green bar indicates peaks which are reduced at least twofold in the mutant cell lines compared to WT. **g** Bar graphs indicating numbers of motifs within the 1975 Sp1 specific peaks in ESC. Separate graphs show all peaks, distal peaks and promoter peaks. The number of peaks is indicated above each bar. **h** Bar graphs indicating numbers of motifs within the 873 Sp1 specific peaks in Flk1+ cells. Separate graphs show all peaks, distal peaks and promoter peaks. The number of peaks is indicated above each bar

Our finding of a residual activity of the Sp1^ΔDBD/ΔDBD^ protein suggested that it may be able to interact with chromatin via other factors, similar to what has been found for SCL/TAL1 [24]. We therefore examined the Sp1 chromatin binding in the A17Lox Sp1^ΔDBD/ΔDBD^ clone and the A17Lox Sp1^−/−^ control clone using global chromatin immunoprecipitation followed by sequencing (ChIP-seq) assays. We used a double crosslinking method of fixation to capture any Sp1 protein that was not directly bound to DNA. Neither clone showed significant levels of binding in either ESC or in Flk1 + cells (Fig. 2a), demonstrating that in spite of its functional activity, the truncated Sp1 protein does not stably interact with chromatin.

To compare the binding of Sp3 with that of Sp1, we performed ChIP-seq for Sp1 and Sp3 in WT A17Lox cells and A17Lox Sp1-disrupted clones. We generated high confidence ChIP-seq peaks for both factors in both ESC and Flk1+ cells by overlapping them with ATAC-seq peaks (Additional file 1: Fig. S2a). We also compared Sp1/3 binding to publicly available datasets to examine whether Sp1/3 factors cooperate with other factors or associate with specific histone modification patterns (Table S1). In A17Lox WT cells, there was a strong overlap in binding sites for Sp1 and Sp3, although there were also unique sites, particularly for Sp3 (Fig. 2b, Additional file 1: Figs. S2b and S2c). Sp1 and Sp3 binding occurred at regions of active chromatin as evidenced by the co-occurrence of histone modifications such as H3K27Ac and H3K4me3 and the absence of H3K27me3 (Fig. 2b). Sp, NFY and YY1 motifs were enriched at shared Sp1/Sp3 binding sites, and corresponding binding of NFY and YY1 at overlapping sites in ESC was observed. Interestingly, we saw enrichment of CTCF motifs and binding at sites which preferentially bound Sp3 but not Sp1. With the exception of Oct4, pluripotency factor binding was not strongly enriched at sites of Sp1/Sp3 binding in ESC (Fig. 2b). In ESC, Sp1 was evenly distributed between promoter and distal sites, while Sp3 was located at a slightly higher number of distal sites (Additional file 1: Fig. S2d). In Flk1+ cells, promoter occupancy increased slightly for both factors (Additional file 1: Fig. S2d).

Comparison of Sp1 and Sp3 binding patterns revealed almost 20,000 shared sites in ESC (Fig. 2c). Sp1 showed a greater than twofold enriched binding compared to Sp3 at 1975 unique sites, while Sp3 occupied 3228 unique sites. The distribution of Sp1/Sp3 peaks was similar in Flk1 + cells; however, fewer Sp1-specific peaks were observed (873, Fig. 2d). Shared and Sp1-specific peaks had higher enrichment for NFY motifs in both ESC and Flk1 + cells, whereas Sp3-specific peaks showed preferential enrichment for CTCF and ETS motifs and surprisingly, an absence of Sp motifs (Additional file 1: Fig. S2e, S2f, S2g, S2h). We and others previously showed that Sp1 binding is enriched at CG islands [3, 15] and Sp1 can bind at multiple motifs in a regulatory region [25]. To examine how Sp1 and Sp3 could co-localise, we mapped the number of motifs within peaks in shared and Sp1-specific peaks (Fig. 2g, h, Additional file 1: Fig. S2i, S2j). In keeping with our previous finding, promoter peaks contained a high frequency of multiple motifs, whereas distal peaks were more likely to have no motif or low numbers of motifs, particularly in Flk1+ cells.

To gain insight into the cooperation of Sp1 and Sp3, we investigated the effect of the lack of Sp1 on Sp3 binding. In general, the absence or mutation of Sp1 had no global effect on the binding of Sp3 (Fig. 2c), demonstrating that binding is not absolutely interdependent. However, we detected a few sites that acquired Sp3 binding in the Sp1-disrupted clones (Fig. 2e). To uncover such sites, Sp3 binding at Sp1-specific peaks was re-ranked according to the strength of Sp3 binding in WT cells and we found 650 newly acquired peaks in the Sp1^ΔDBD/ΔDBD^  cells and 595 newly acquired peaks in the Sp1^−/−^ cells (Fig. 2e). This effect was less pronounced in Flk1 + cells with only 57 newly acquired peaks in the Sp1^ΔDBD/ΔDBD^ cells (Fig. 2f). This result would suggest that in ESC, Sp3 can compensate for loss of Sp1 at a subset of binding sites but this effect may not be maintained throughout differentiation since fewer sites in Flk1+ cells acquired de novo Sp3 binding in the absence of Sp1.

### Sp1 and Sp3 mutant ESC exhibit common and unique features during hematopoietic differentiation

**Fig. 3:**
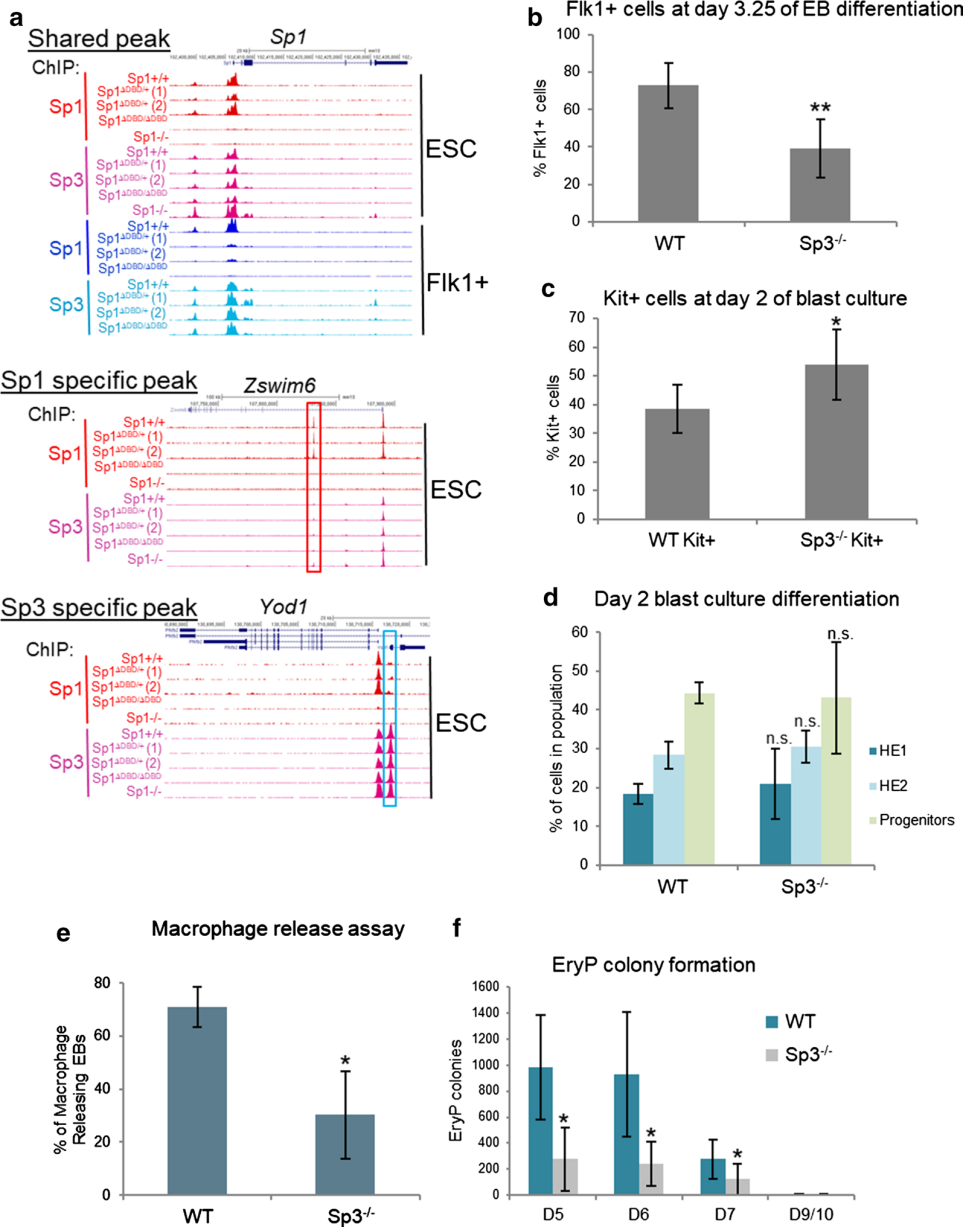
Sp3^−/−^ ES cells exhibit a defect in hematopoietic differentiation. **a** Genome browser screenshots depicting examples of shared and unique binding sites. Top panel: *Sp1* is an example of an Sp1/Sp3 shared promoter binding site; middle panel: *Zswim6* is an example of an intragenic Sp1 specific peak (highlighted by the red box); bottom panel: *Yod1* is an example of an Sp3 specific promoter peak (highlighted by the blue box). **b** Flk1 FACS staining on WT and Sp3^−/−^ cells at day 3.25 of EB differentiation (*n* = 5, **indicates *p* < 0.01). **c** Kit FACS staining of WT and Sp3^−/−^ cells at day 2 of blast culture (*n* = 4, *indicates *p* < 0.05). **d** Percentage cell populations from WT and Sp3^−/−^ cells at day 2 of blast culture (*n* = 4, n.s. indicates not statistically significant). **e** Percentage of macrophage-releasing EB in a macrophage release assay comparing WT and Sp3^−/−^ cells (*n* = 4, *indicates *p* < 0.05). **f** Number of primitive erythroid colonies in an EryP colony formation assay comparing WT and Sp3^−/−^ cells (*n* = 4, *indicates *p* < 0.05)

**Fig. 4:**
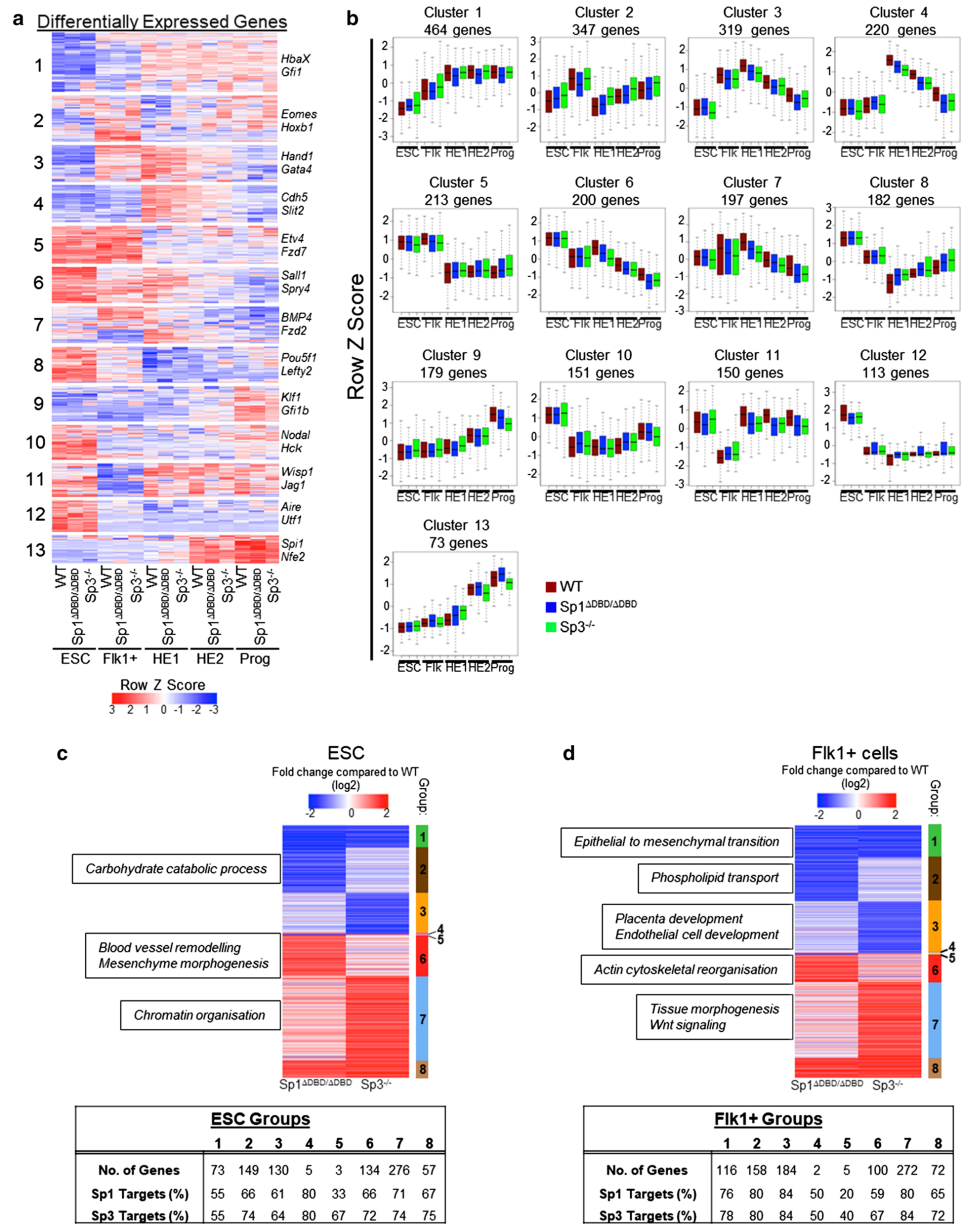
Deregulated gene expression in Sp1^ΔDBD/ΔDBD^ and Sp3^−/−^ cell populations. **a** Hierarchical clustering of differentially regulated genes between WT, Sp1^ΔDBD/ΔDBD^ and Sp3^−/−^ cell populations from each stage of the differentiation series according to the row Z score. Data were separated into 13 clusters, and the number of the cluster is shown to the left of the heat map. Representative genes from each cluster are shown to the right of the heat map. **b** Box plots representing the Z score of gene expression of each individual cluster from (**a**). **c** Heat map representing the grouping analysis of differentially regulated genes for Sp1^ΔDBD/ΔDBD^ relative to WT and Sp3^−/−^ relative to WT in ESC. The coloured sidebar indicates the 8 clusters assigned according to the changes in gene expression. GO terms for selected groups are shown to the left of the heat map. The table beneath shows the number of deregulated genes in each group and the number of Sp1 and Sp3 target genes within each cluster. **d** Heat map representing the grouping analysis of differentially regulated genes for Sp1^ΔDBD/ΔDBD^ relative to WT in Flk1+ cells, and Sp3^−/−^ relative to WT in Flk1+ cells. The coloured sidebar indicates the 8 clusters assigned according to the changes in gene expression. GO terms for selected groups are shown to the left of the heat map. The table beneath shows the number of deregulated genes in each group and the number of Sp1 and Sp3 target genes within each cluster

We next investigated the contribution of Sp1 and Sp3 to hematopoietic specification (Fig. 3) and gene expression (Fig. 4). Although Sp1 and Sp3 share the majority of their binding sites as exemplified at the *Sp1* locus, they also demonstrate specific binding (Fig. 3a, top panel). The gene encoding zinc finger domain-containing protein *Zswim6* has a shared Sp1/Sp3 site at the promoter in addition to an intergenic Sp1-specific peak, whereas the *Yod1* deubiquitinase gene has a Sp3-specific peak at the promoter. Since Sp3 appeared to compensate at some but not all Sp1 binding sites in the absence of Sp1 (Fig. 2e), we examined in more detail how blood cell development from ESC was affected in the absence of Sp3. Using an E14 ESC line lacking Sp3 [8] (Additional file 1: Figs. S3a, b), we found that these cells generate a reduced number of Flk1+ cells after 3.25 days of EB formation (Fig. 3b), although the phenotype was less pronounced when compared with Sp1^ΔDBD/ΔDBD^ and Sp1^−/−^ cells (Fig. 1). Sp3−/− Flk1+ cells produced more Kit-expressing cells in blast culture but showed similar proportions of hemogenic endothelium (HE) and progenitors compared to WT cells (Fig. 3b, d; Additional file 1: Fig. S3d). In common with E14 Sp1^ΔDBD/ΔDBD^ ESC (Fig. 1), E14 Sp3^−/−^ ESC showed defective macrophage production in macrophage release assays (Fig. 3e, Additional file 1: Fig. S3d).

Production of primitive erythrocytes was severely impaired in E14 Sp3^−/−^ cells compared to WT cells (Fig. 3f), whereas we previously found that E14 Sp1^ΔDBD/ΔDBD^ ESC showed only delayed production of primitive erythrocytes [3]. We attempted to produce an ES cell line with inducible Cre-responsive conditional deletion of both Sp3 and the Sp1 DBD. A mouse carrying these alleles shows severe macrothrombocytopenia after megakaryocyte-specific deletion of these genes [26]. In spite of multiple attempts, we did not obtain any clones carrying both deletions, indicating that ESC maintenance requires the presence of intact versions of either Sp1 or Sp3, again demonstrating that the extent of requirement for Sp1/Sp3 proteins is developmental stage specific with defects being more severe at early developmental stages.

To investigate whether the mutation of Sp1 and Sp3 differentially affected gene expression at the various developmental stages, we performed RNA-seq with E14 ESC, as well as with sorted Flk1+ , HE1, HE2 and progenitor cells from E14 WT, E14 Sp1^ΔDBD/ΔDBD^ and E14 Sp3^−/−^ cells (Additional file 1: Fig. S4a). Hierarchical clustering of the row Z scores from all expressed genes revealed the expected dynamic changes in gene expression across the differentiation stages for all cell lines (Additional file 1: Fig. S4b). This result indicates that the majority of differentiation-associated gene expression changes were unaffected by the mutations. To examine genes whose expression levels were differentially affected between WT cells and either E14 Sp1^ΔDBD/ΔDBD^ or E14 Sp3^−/−^ cells in at least one of the differentiation stages (Additional file 1: Fig. S4c, S4d), we used covariance analysis (Fig. 4a and Additional file 2: Dataset 1 for gene lists). Representative genes from each cluster are shown (Fig. 4b). These patterns of expression clustered together irrespective of the cell line, and average gene expression across clusters was again broadly similar between the cell lines (Additional file 1: Fig. S4b). However, this was not true for all genes. Within cluster 1, a sub-cluster of 92 genes showed increased expression in the Sp3^−/−^ cells compared to WT and Sp1^ΔDBD/ΔDBD^ cells (Additional file 2: Dataset 1 for gene lists). Within cluster 4, 35 genes showed reduced expression in Sp1^ΔDBD/ΔDBD^ cells relative to both WT and Sp3^−/−^ cells (Additional file 1: Fig. S4e).

To examine the direction of cell type-specific gene expression changes in the different types of mutant ESC or Flk1+ cells in more detail, we grouped genes according to whether they were up- or down-regulated at least twofold in either E14 Sp1^ΔDBD/ΔDBD^ or E14 Sp3^−/−^ cells compared to E14 WT cells (Fig. 4c, d and Additional file 3: Dataset 2). Cells were separated into 8 different groups for each cell type. Despite the large overlap in DNA-binding sites (Fig. 2), Sp1 and Sp3 mutant ESC showed mostly independent gene expression changes, with only a few shared up- and down-regulated genes (Fig. 4c, d, Groups 1 and 8). Very few genes showed completely reciprocal changes in expression when compared with WT (Fig. 4c, d, Groups 4 and 5). The majority of deregulated genes were targets of both Sp1 and Sp3 with only a minority of genes being unique Sp1 or Sp3 targets (Additional file 1: Fig. S4f, S4g).

In summary, these results demonstrate that (1) the overall cell differentiation trajectory up to the progenitor stage is largely unaffected in Sp1^ΔDBD/ΔDBD^ and Sp3^−/−^ cells, and (2) the interplay of Sp1 and Sp3 at shared sites is context dependent with only a small proportion of genes changing expression the same direction in the absence of either Sp1 or Sp3.

### Single-cell RNA-seq analysis reveals  a deviation from normal differentiation patterns in Sp1^ΔDBD/ΔDBD^ blast culture cells

Our previous gene expression microarray analysis of the effect of the E14 Sp1^ΔDBD/ΔDBD^ mutation on gene expression showed that it was progressively deregulated compared to WT cells as differentiation progressed, with more and more genes displaying perturbed expression [3]. Interestingly, when we subjected these data to a principal component analysis, we noticed that the data from E14 Sp1^ΔDBD/ΔDBD^ cells showed the same differentiation trajectory up to progenitor cells, but were characterised by a third, unknown component. This was confirmed when we used RNA-seq and unbiased clustering analysis (Additional file 1: Fig. S5A, data not shown). We next investigated the nature of this third component. However, a significant problem with the analysis of bulk cell populations is that it is possible to obtain trends in gene expression, but even with FACS-sorted populations there can be significant cellular heterogeneity. It was therefore unclear whether the progressive deviation in gene expression was a faithful reflection of the expression pattern within individual cells, or a consequence of increased cellular heterogeneity within the sorted populations.

**Fig. 5:**
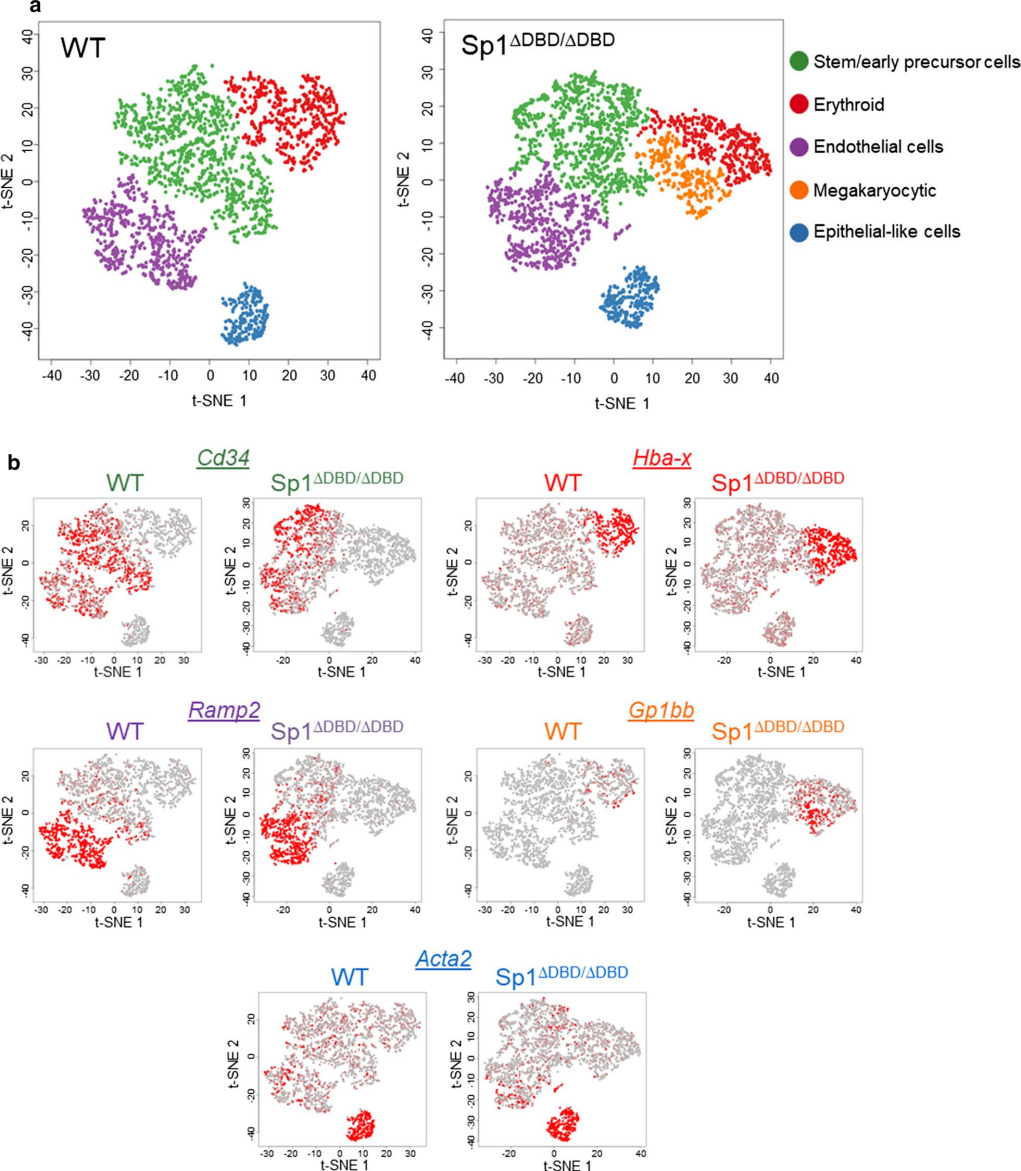
Identification of different cell populations in differentiating WT and Sp1^ΔDBD/ΔDBD^ cells. **a** t-SNE visualisation of the indicated cell populations within Sp1^ΔDBD/ΔDBD^ Kit+ sorted cells from day 2 of blast culture. The left panel shows WT cells; right panel shows Sp1^ΔDBD/ΔDBD^ cells. Each dot represents the transcriptome of a single cell, colour coded according to the assigned cellular identity as shown to the right of the panels. **b** Expression of selected marker genes superimposed onto the different clusters for WT and Sp1^ΔDBD/ΔDBD^ cells

To answer this question, we used the 10x  Genomics Chromium single-cell RNA-seq system to investigate the heterogeneity in gene expression in day 2 blast cultures obtained from E14 WT and E14 Sp1^ΔDBD/ΔDBD^ ESC. At this developmental stage, we can isolate cells representing multiple differentiation stages from the same culture. Cells were sorted for surface Kit expression prior to loading cells on the instrument (Additional file 1: Fig. S5b). Sequencing data from more than 2000 cells for each sample were used for downstream analysis (Additional file 1: Fig. S5c). We used t-distributed stochastic neighbour embedding (t-SNE) analysis to visualise clusters with similar gene expression profiles from E14 WT and E14 Sp1^ΔDBD/ΔDBD^ cells (Fig. 5a). Four distinct clusters were identified in both samples with the help of typical marker genes listed in Additional file 4: Dataset 3, resembling early progenitor cells (green), erythroid cells (red), endothelial cells (purple) and epithelial-like cells of unknown origin (blue). However, an additional megakaryocytic cluster was found in the E14 Sp1^ΔDBD/ΔDBD^ cell population (orange), and the other clusters displayed different shapes. A heat map showing the specificity of marker genes for each individual cluster is shown in Additional file 1: Fig. S5d. Gene expression for a selection of these markers was mapped onto the cell clusters (Fig. 5b). For example, *Cd34* was predominantly expressed in the cluster resembling stem and early precursor cells, whereas *Hba*-*X* was mainly expressed in erythroid cells and an increased number of E14 Sp1^ΔDBD/ΔDBD^ cells expressed the megakaryocyte marker *Gp1bb*  (Fig. 5b).

**Fig. 6:**
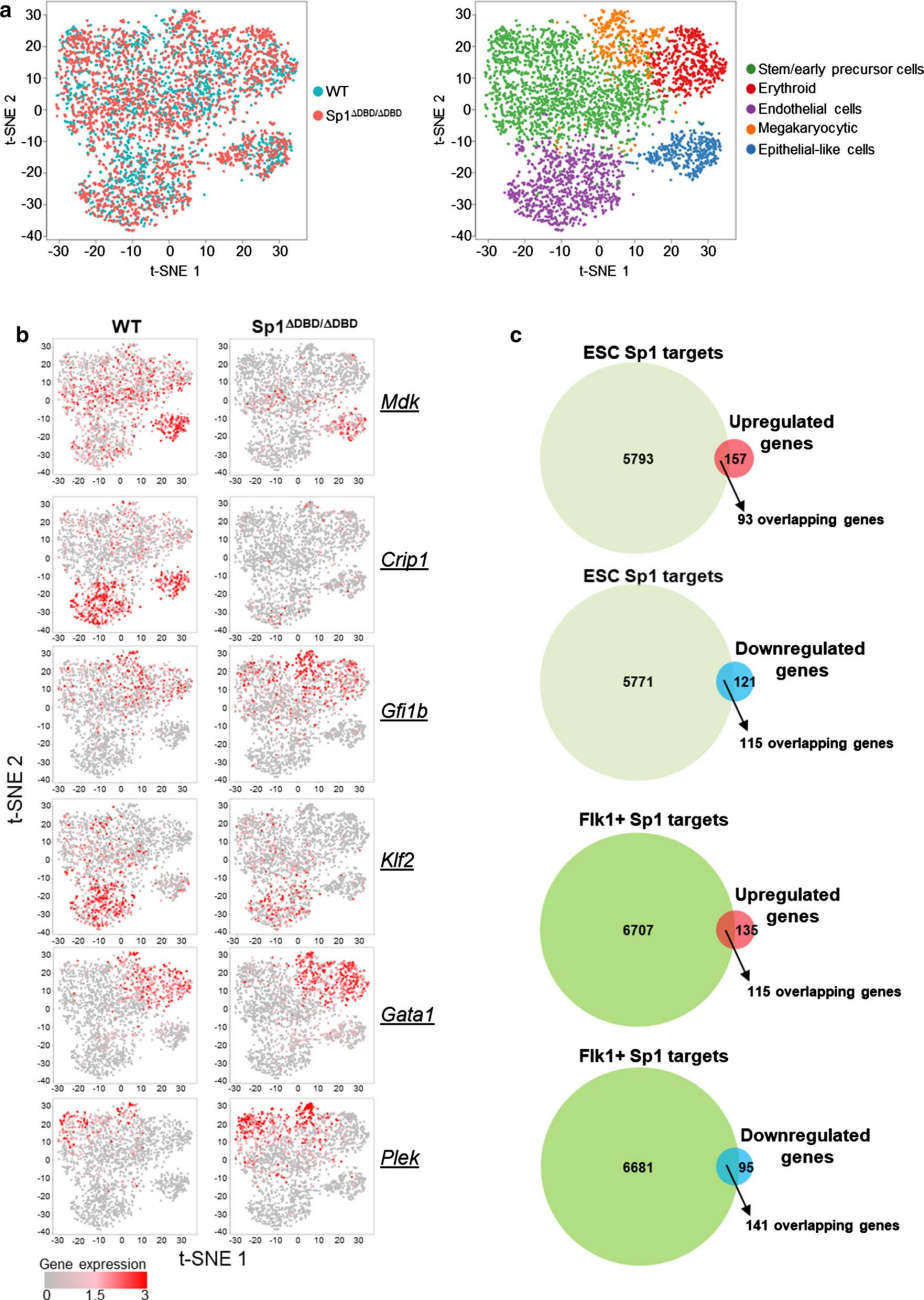
Sp1^ΔDBD/ΔDBD^ cells contain an additional megakaryocyte cluster as compared to WT cells. **a** t-SNE visualisation of combined WT and Sp1^ΔDBD/ΔDBD^ populations. Each dot represents a single cell. In the left panel, dots are coloured to represent the genotype as indicated. In the right panel, dots are coloured according to the cell identity as indicated. **b** The expression level of selected differentially expressed genes was superimposed onto the t-SNE plots shown in Fig. 6a. Gene names are indicated to the right of the panel. **c** Venn diagrams representing the overlap between Sp1 ChIP-seq targets in ESC (light green circles) or Flk1+ cells (dark green circles) with up-regulated genes (red circles) or down-regulated genes (blue circles) determined from single-cell differential gene expression analysis. The number of genes for each group is indicated

We next performed differential gene expression analysis to explore in more detail the differences behind the deregulation of E14 Sp1^ΔDBD/ΔDBD^ differentiation. t-SNE plots showing both E14 WT and E14 Sp1^ΔDBD/ΔDBD^ cells were coloured according to the genotype of the cell line (Fig. 6a, left panel) or according to cell type cluster (Fig. 6a, right panel), illustrating the spread of the individual cells across the different clusters. These results also demonstrate that genes specific for particular cell types are expressed in the relevant cluster and that the same clusters are present in WT and mutant cells (Fig. 6b). We then overlaid the relative gene expression for selected differentially expressed genes onto these plots thus allowing us to visualise the extent of variation of an individual gene between the cell lines across the different clusters; several examples are shown in Fig. 6b and Additional file 1: Fig. S6b. One example is the growth factor Midkine (*Mdk*) which was found to be an Sp1 target in human glioma cells [27], and from our single cell data showed substantial down-regulation across all E14 Sp1^ΔDBD/ΔDBD^ cell clusters, although some individual cells retained strong expression particularly in epithelial cells. Normalised FPKM values from RNA-seq of the bulk populations demonstrated only a modest down-regulation of *Mdk* at all stages except ESC highlighting the importance of single-cell data to uncover such differences (Additional file 1: Fig. S6c). *Mdk* has been proposed as a regulator of the epithelial–mesenchymal transition (EMT) during mouse embryogenesis. Furthermore, it has been implicated in Notch signalling pathways and as an antagonist of vascular endothelial growth factor (VEGF) suggesting that it may play a role in development [28–30]. Cysteine-rich intestinal protein 1 (*Crip1*) was also proposed as a mediator of EMT, in this case in human cervical carcinoma via the Wnt signalling pathway [31]. We found that expression of *Crip1* was strongly down-regulated in E14 Sp1^ΔDBD/ΔDBD^ cells, particularly in endothelial and epithelial clusters (Fig. 6b). This finding correlated with changes seen in the bulk RNA-seq data, and interestingly, differences in *Crip1* gene expression were evident as early as the ESC stage of development (Additional file 1: Fig. S6c). *Klf2* is expressed in murine vascular endothelial cells during early embryogenesis, and mice lacking *Klf2* display compromised vascular integrity and die in utero as a result of haemorrhage [32]. E14 Sp1^ΔDBD/ΔDBD^ endothelial cells show reduced expression of *Klf2* suggesting a potential defect in the formation or function of the hemogenic endothelium (Fig. 6b). A comparison of genes deregulated in E14 Sp1^ΔDBD/ΔDBD^ in Kit + day 2 of blast culture cell populations with Sp1 target genes in ESC and Flk1+ cells indicates that a proportion of the genes were already Sp1 targets early in development (Fig. 6c). In summary, these results confirm that while differentiation can progress in E14 Sp1^ΔDBD/ΔDBD^ cells, normal Sp1 function is required to maintain the proper timing of lineage specification.

**Fig. 7:**
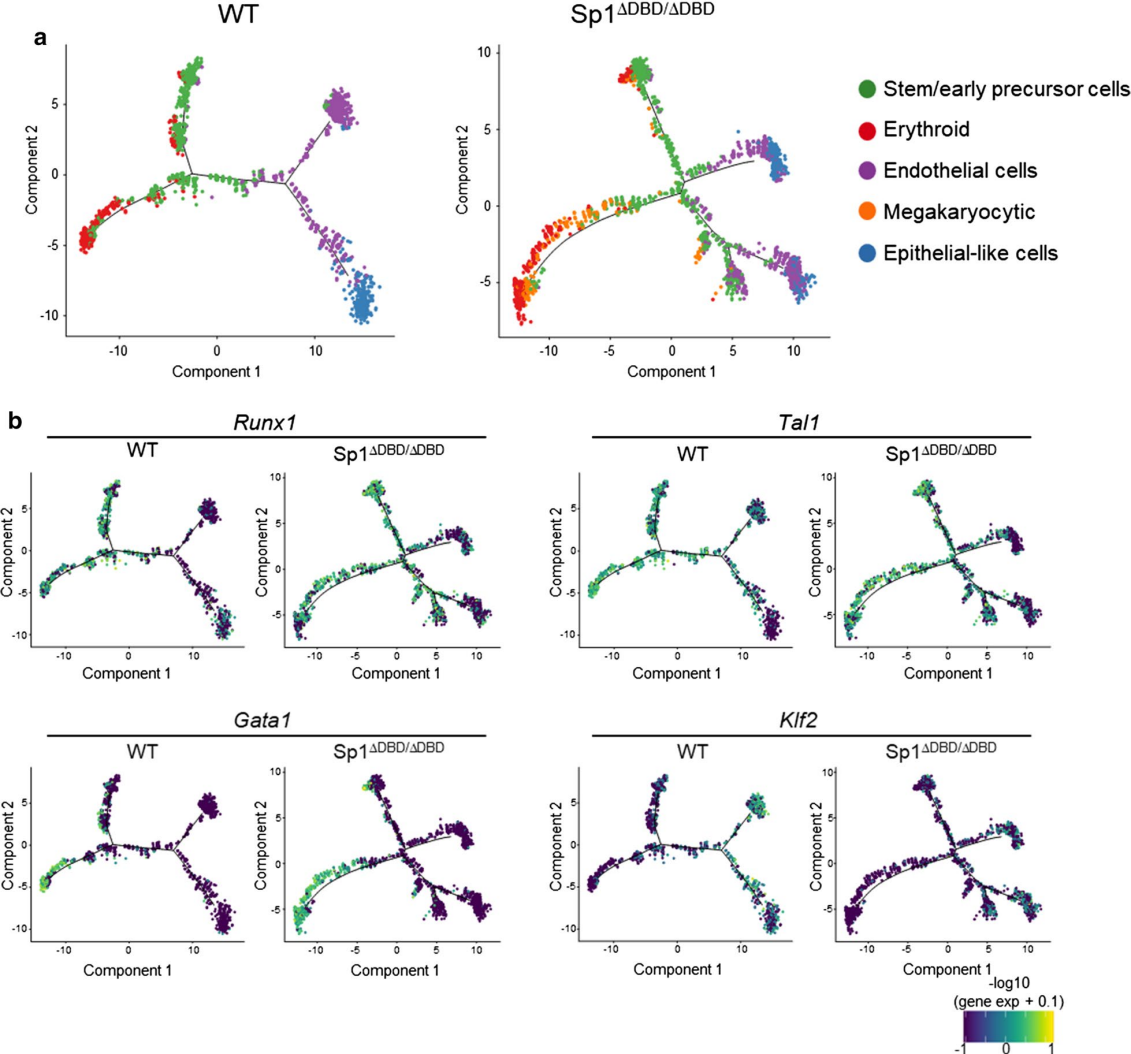
Absence of functional Sp1 results in highly disordered differentiation trajectories. **a** Pseudotime trajectory plots of WT and Sp1^ΔDBD/ΔDBD^ Kit + sorted cells from day 2 of blast culture. Each dot represents the transcriptome of a single cell, colour coded according to the assigned cellular identity as shown to the right of the panels. **b** Expression patterns of selected TFs projected on the trajectory plots for WT and Sp1^ΔDBD/ΔDBD^ from day 2 of blast culture

Our cell populations represent a dynamic differentiation system, and analysis of the single-cell transcriptome data allows inference of differentiation trajectories based on gene expression from individual cells. To this end, we performed pseudotime ordering to identify the trajectory of differentiation in E14 WT and E14 Sp1^ΔDBD/ΔDBD^ cells. Here, individual cells are displayed along these trajectories based on the cluster they represent (Fig. 7a). In E14 WT cells, cell clusters were associated with two distinct trajectories with cells following a hematopoietic and an endothelial differentiation trajectory which is expected for the mixed population of cells developing at this stage of differentiation [19]. In contrast, differentiation trajectories in Sp1^ΔDBD/ΔDBD^ cells were strongly disordered with the direction of the trajectory for each cell fate decision being preserved, but with more branch points, indicating that the timing of differentiation was disturbed, thus explaining the third PCA component. In addition, we see an enhanced development of megakaryocytes. This type of disordered differentiation was confirmed when we overlaid the expression patterns of individual genes along the projected trajectories (Fig. 7b, Additional file 1: Fig. S7a). For both cell lines, expression of *Tal1* was widespread throughout all lineages but absent from epithelial-like cells, consistent with its known role in hemangioblasts and in blood formation [33]. *Runx1* was expressed in committed hematopoietic cells, and so was the RUNX1 target *Spi1* in both E14 WT cells and E14 Sp1^ΔDBD/ΔDBD^ cells. The TGFβ receptor component Endoglin (*Eng*) showed highest expression within the endothelial cell cluster, consistent with its role in the formation of both blood cells and blood vessels [34]. In Sp1^ΔDBD/ΔDBD^ cells, *Eng* expression was spread across multiple branch points with hematopoietic cells branching off. Taken together, our data show that Sp1 is not involved in cell fate decisions, but strongly influences at which time point cell fate decisions are executed.

## Discussion

A large number of promoters and distal elements contain binding sites for two members of the Sp family of transcription factors, Sp1 and Sp3. These proteins have been studied for decades, and their important role in development has been established, but their precise function in regulating specific developmental pathways is still largely unknown. This study used systems-level analysis to address this issue and answer a number of fundamental questions of how Sp1 and Sp3 are involved in driving hematopoietic specification.

### Sp1 and Sp3 cooperate to generate blood cells

Our study finds that both Sp1 and Sp3 are important for correct hematopoietic specification whereby the complete lack of Sp1 is incompatible with differentiation. We show that the original Sp1 knockout [7] which introduced a truncated Sp1^ΔDBD/ΔDBD^ protein constitutes a hypomorphic version of this factor with residual activity. The complete Sp3 knockout (Sp3^−/−^) also has a blood differentiation phenotype and shares some features of the Sp1 hypomorphic phenotype including reduced Flk1 expression and reduced macrophage production from EBs. However, compared to Sp1^ΔDBD/ΔDBD^ cells the phenotype is weaker which is in agreement with the observations in mice [8]. While the simultaneous mutation of both *Sp1* and *Sp3* is incompatible with ESC maintenance, either is dispensable for the establishment of a correct, cell type-specific chromatin accessibility pattern. This result demonstrates that cell fate decisions are not driven by Sp1 and Sp3 but by tissue-specific factors. The majority of Sp1 and Sp3 sites overlap, and with some exceptions, Sp3 binding is not dependent on the presence of Sp1. However, both proteins need to cooperate for normal development to occur, as shown by the ability of cells to differentiate up to a certain point when the Sp1^ΔDBD^ protein is present together with Sp3.

Previous studies provided some indications for such DBD-independent interactions between Sp-factors. The Sp family member Sp2 was found to bind to chromatin through association with NFY and independent of its DBD [35]. However, note that this protein differs from other Sp family members since it has only one glutamine-rich domain instead of the 2 found in Sp1 and Sp3, and it does not recognise the conventional Sp binding motif [36]. Sp1^ΔDBD/ΔDBD^ mice and ESC retain the N-terminal portion of the Sp1 protein [7]. Such Sp1 deletion mutants promote synergistic activation of promoter constructs when co-transfected with full-length Sp1 constructs or with Sp4 in Drosophila SL2 cells lacking endogenous Sp1 activity [25]. The DBD mutant has been described as a super-activator, and it was observed to contribute to multimeric Sp1 complexes in in vitro DNA-binding studies [37]. Note that many Sp1/3 binding sites occur in CG island promoters that contain multiple Sp binding motifs, providing a surface for multiple protein–protein interactions. We speculate that the activity of the Sp1^ΔDBD^ protein to rescue part of the hematopoietic differentiation program is due to a transient interaction with other factors in chromatin that impacts on gene expression. Such transient but functional interactions that leave their mark behind in chromatin have been observed previously at distal elements [38, 39]. However, additional experiments are required to address this issue.

### The full activity of Sp1 is required for robust and coordinated hematopoietic specification

Developmental pathways are regulated by the interplay of external signals and intracellular factors and are normally highly robust, with cells executing cell fate decisions in a highly coordinated fashion within the developing organism. Many tissue-specific TFs have been shown to be essential for the execution of specific cell fates. For example, erythroid and megakaryocyte development requires the presence of GATA1 [40] and SCL/TAL1 is essential to form all blood cells [41]. However, the mechanism by which such factors actually execute cell fate decisions is still under intensive investigation and debate. The recent development of techniques that determine the pattern of gene expression in single cells allowed the discovery of previously hidden cellular heterogeneity within populations as well as lineage infidelity of gene expression within individual cells and, most importantly, made it possible to follow differentiation trajectories (reviewed in [42]). For example, single-cell profiling of hematopoietic stem- and progenitor populations has revealed a continuum of hematopoietic differentiation rather than discrete stages of development [43] and highlighted how such decisions are perturbed in the absence of specific TFs [44].

In the studies published so far, the perturbation of tissue-specific factors generally led to a loss or defect of a specific differentiation trajectory such as the complete loss of cells undergoing blood development in *Tal1*-null embryos [45] or *Tal1*-null cells from chimaeric embryos [46] or the inability of cells from *Cebpa-*null embryos to enter myelopoiesis [44]. Our results point to an important difference between the role of Sp1/3 and tissue-specific factors since the absence of stable chromatin binding of the Sp1^ΔDBD^ protein makes differentiation inherently unstable without disturbing the general trajectories of early cell fate decisions. Such increased destabilisation manifests itself in an increased heterogeneity of gene expression within sorted cell populations [3]. Our single-cell analysis resolved this heterogeneity into a profound disturbance of the coordination of cell fate decisions within the developing population. This disturbance led to the existence of multiple branch points following a similar differentiation trajectory, i.e. the timing and coordination of a specific cell fate decision within the blood specification trajectory were disturbed. In addition, we observed an increase in onset of megakaryopoiesis at the expense of definitive erythropoiesis. The expression of genes encoding tissue-specific TFs such as *RUNX1*, *GFI1* or *GATA1*, capable of regulating such cell fate decisions, followed the path of the disturbed trajectory in Sp1^ΔDBD/ΔDBD^ cells. This behaviour indicates that while these genes are expressed in the correct cells and cell fate decisions are preserved, the robustness of this developmental pathway is diminished. This notion is also consistent with the absence of major differences in cell type-specific chromatin states of WT and Sp1^ΔDBD/ΔDBD^ cells, meaning that within the developing population the correct chromatin transitions between ESC and FLK1+ cells take place, and this holds true for both promoter and distal sites (data not shown). Since Sp1 sites are enriched in promoters, we therefore propose that Sp1 and Sp3 are involved in setting up a stable promoter structure that is capable of interacting with distal elements in a coordinated fashion. In Sp1^ΔDBD/ΔDBD^ cells, these interactions are destabilised, introducing a stochastic element into differentiation. As the cells approach the terminally differentiated state, the accumulation of deregulation events and further destabilisation prevent the execution of differentiation past a certain point.

## Conclusions

Our results explain why the phenotype of the original Sp1^ΔDBD/ΔDBD^ mouse is highly heterogeneous with development blocked at various early stages [7] and why the introduction of the same allele in late precursor cells such as myeloblast/monocytes has no effect on differentiation [3]. Sp1/Sp3 do not co-localise with a specific class of factors at distal elements but cooperate with distal elements bound by factors specific for each cell type. Consistent with this notion, the defect of Sp1 leads to multiple abnormalities in many tissues in the mouse. Our data are consistent with the idea that Sp1 and Sp3 are essential elements of coordinated and robust developmental processes. We propose that other general and ubiquitously expressed transcriptional regulators generate similar phenotypes with development becoming uncoordinated and unstable, thus setting the stage for further aberrations such as developmental defects and cancer.

## Methods

### Mouse ESC culture

WT, Sp1^ΔDBD/ΔDBD^ and Sp3^−/−^ cells [3, 7, 8] as well as A17 2Lox ESC (a gift from Michael Kyba) were maintained in ESC maintenance media: DMEM (Sigma D6546), supplemented with 15% FCS, 100 units/ml penicillin and 100 µg/ml streptomycin, 1 mM sodium pyruvate, 1 mM glutamine, 0.15 mM MTG, 25 mM HEPES buffer, 10^3^ U/ml ESGRO (Millipore), 1x non-essential amino acids (Sigma) on primary mouse embryonic fibroblast (MEF) feeder cells. ESC were grown in the absence of feeder cells for two passages prior to differentiation.

### In vitro differentiation of mouse ESC

In vitro differentiation of ESC was performed as previously described [3, 47, 48]. More information can be found in Additional file 1.

### Cell population sorting

HE1, HE2 and progenitor populations for RNA-seq and ATAC-seq were prepared at day 2 of blast culture by cell sorting according to surface marker expression. Floating and adherent cells were harvested and combined before staining with KIT-APC (BD Pharmingen 553356), CD41-PE-Cy7 (ebioscience 25-0411) and Tie2-PE (ebioscience 12-5987) antibodies in MACS buffer. After washing, cells were separated on a FACS Aria cell sorter (BD Biosciences) into HE1 (KIT+, CD41−, Tie2+); HE2 (KIT+, CD41+, Tie2+) and progenitor (KIT+, CD41+, Tie2−) cell populations according to surface marker expression as indicated.

### CRISPR deletion of Sp1 DBD in ESC

Guide RNA sequences targeting the Sp1 DNA-binding domain (Additional file 1: Fig. S1a) were designed using the CRISPR Design Tool by the Zhang laboratory [49, 50]. CRISPR guide 1: 5′-TATACTTTGCCGCATCCT; CRISPR guide 2: 5′-TTGCATCCCGGGCTTAGT. Annealed double-stranded oligos with compatible ends were cloned into plasmid pSpCas9(BB)-2A-GFP (PX458) (a gift from Feng Zhang—Addgene #48138) at the BbsI site [21, 51].

A17 2Lox murine ESC were transfected with two pX458 plasmids, expressing either CRISPR guide 1 or CRISPR guide 2 using the Amaxa P3 4D-Nucleofector kit (Lonza), according to manufacturer’s guidelines. Transfected cells were seeded onto gelatine-coated dishes. GFP expressing cells were purified by cell sorting, and single cells were seeded into 96-well plates coated with MEFs. GFP + single cell clones were expanded and tested for disruption of the Sp1 DBD by PCR of genomic DNA using the following primers: FW-5′-TGGCACACATACCTTTAATCCT and Rev-5′-ACCTGGGATGAGATAAATGCTG. The product obtained from WT DNA was 1564 bp, whereas successful deletion of the target region resulted in a product of 496 bp.

### Overexpression of Sp1 in ESC CRISPR clones

Sp1^ΔDBD/ΔDBD^ and Sp1^−/−^ ES cells were co-transfected with a PiggyBac (PB) transposase expression vector (PL623) [52] and a PiggyBac vector containing the coding sequence of human Sp1 (PB-PGK-SP1-2A-mCherry) using Amaxa nucleofection. Cells were sorted for mCherry expression 48 h after transfection. Sorted cells were grown on MEFs for approximately 5 days, after which individual clones were picked and expanded. Full-length Sp1 expression was then assayed by qPCR analysis and Western blotting. Once positive clones were identified, the capacity of clones to express Flk1 during in vitro differentiation of EBs was measured.

### Macrophage release assay

Macrophage release assays were performed essentially as previously described [3]. Briefly, ESC were trypsinised and allowed to form EB by plating in base methylcellulose (Stem Cell Technologies M3134) supplemented with 10% FCS, 100 units/ml penicillin and 100 µg/ml streptomycin, 1 mM glutamine, 0.15 mM MTG, 10 µg/ml insulin (Sigma), 5% IL-3 conditioned media, 100 units/ml IL-1 (Peprotech) and 25 ng/ml recombinant mouse M-CSF (R&D Systems), at a cell concentration previously determined to give similar numbers of EB. EB were counted after at least 14 days, and the number of EB surrounded by a halo of macrophages were determined.

### EryP assay

EryP assays were performed essentially as previously described [3], and further details can be found in Additional file 1.

### ChIP-seq

ChIP-seq libraries were prepared using the Kapa Hyper Prep kit according to manufacturer’s instruction and as previously described [53]. More information on ChIP and ChIP-seq can be found in Additional file 1.

### RNA-seq library preparation

RNA was isolated from cells at each stage of the differentiation protocol as described previously (ESC, Flk1+, HE1, HE2 and progenitor) [3]. RNA-seq libraries were prepared from two biological replicates for each sample as described previously [53]. The Tru-Seq Stranded Total RNA kit (Illumina) was used to prepare libraries according to manufacturer’s instructions. Libraries were sequenced in a pool of 12 indexed libraries using a NextSeq 500/550 High Output Kit v2 (150 cycles) for paired-end sequencing (Illumina) at the Genomics Birmingham sequencing facility.

### ATAC-seq

ATAC-seq was performed in duplicate, on 50,000 cells per sample isolated from each stage of the differentiation protocol (ESC, Flk1+ cells). ATAC-seq libraries were prepared essentially as described [54, 55], and more detail can be found in Additional file 1. Libraries were sequenced in a pool of 12 indexed libraries using a NextSeq 500/550 High Output Kit v2 (150 cycles) for paired-end sequencing (Illumina) at the Genomics Birmingham sequencing facility.

### Single-cell RNA-seq

Single-cell suspensions of E14 WT and E14 Sp1^ΔDBD/ΔDBD^ cells were sorted for Kit expression at day 2 of blast culture prior to loading 4000 single cells of each population on a Chromium Single Cell Instrument (10X Genomics). Library generation for single-cell RNA-seq was performed as a service by the Genomics Birmingham Sequencing Facility using the Chromium Single Cell 3′ Library and Gel Bead Kit v2 (10X Genomics). Libraries were subjected to paired-end sequencing on an Illumina NextSeq 500/550 sequencer according to 10X Genomics recommended cycle parameters.

### Protein gels and western blotting

Protein extracts in Laemmli buffer were separated on 4–20% gradient pre-cast gels (Bio-Rad) and transferred to nitrocellulose using either Turbo transfer packs (Bio-Rad) with a semi-dry blotter (Bio-Rad) or by using wet transfer to nitrocellulose in 0.1 mM CAPS/10% methanol transfer buffer. Blots were blocked with 4% milk powder in 0.1% TBS-Tween prior to antibody incubation. Antibodies used were: Sp1, Millipore 07-645; Sp3, Santa Cruz sc365220; GAPDH, Abcam ab8245; H3, Abcam ab1791.

### Bioinformatic analysis

Details about the bioinformatic analysis of genome-wide data sets are described in Additional files 1 and 5.

## Additional files

**Additional file 1.** Supplemental Methods and Figures.

**Additional file 2.** Dataset 1 - Covariance analysis of RNA-seq data.

**Additional file 3.** Dataset 2 - Grouping analysis of RNA-seq data and ChIP-seq data.

**Additional file 4.** Dataset 3 - scRNA-seq cell cluster markers

**Additional file 5.** Sequencing data list

## Data Availability

All datasets generated in this study have been deposited at the NCBI gene expression omnibus (https://www.ncbi.nlm.nih.gov/geo/) under accession numbers GSE126497 and GSE126501. A Reporting Summary for this article will be available as a Supplemental Information file. All other data supporting the findings of this study are available from the corresponding author upon request.
