# Supplementary material for: Robust hematopoietic specification requires the ubiquitous Sp1 and Sp3 transcription factors
Source: Epigenetics Chromatin. 2019 Jun 4;12:33. doi: 10.1186/s13072-019-0282-9 (PMC6547542; doi:10.1186/s13072-019-0282-9)
Supplement: Supplementary file 1 — Additional file 1. Supplemental Methods and Figures. [file 13072_2019_282_MOESM1_ESM.docx]

**Robust hematopoietic specification requires the ubiquitous Sp1 and Sp3 transcription factors**

Jane Gilmour, Leigh O’Connor, Christopher P. Middleton, Peter Keane, Nynke Gillemans, Jean-Baptiste Cazier, Sjaak Philipsen, Constanze Bonifer.

**Supplemental Information**

**Table of Contents:**

**List of Supplemental Datasets**

**List of Supplemental Tables**

**Supplemental Figures**

**Supplemental Experimental Procedures**

**Supplemental References**

**Supplemental Datasets:**

Supplemental Dataset S1: Covariance analysis

File: Supplemental Dataset S1_Covariance_clusters.xlsx

Supplemental Dataset S2: RNA-seq Grouping analysis

File: Supplemental Dataset S2_Grouping analysis_ChIP targets.xlsx

Supplemental Dataset S3: scRNAseq Cell Cluster Markers - Gene Lists

File: Supplemental Dataset S3_scRNAseq_CellClusterMarkers_GeneLists.xlsx

Supplemental Dataset S4: Sequencing data

File: Supplemental Dataset S4_Sequencing data.ppt

**Supplemental Tables**

Supplemental Table 1: Public datasets

**Supplemental Figures**

**
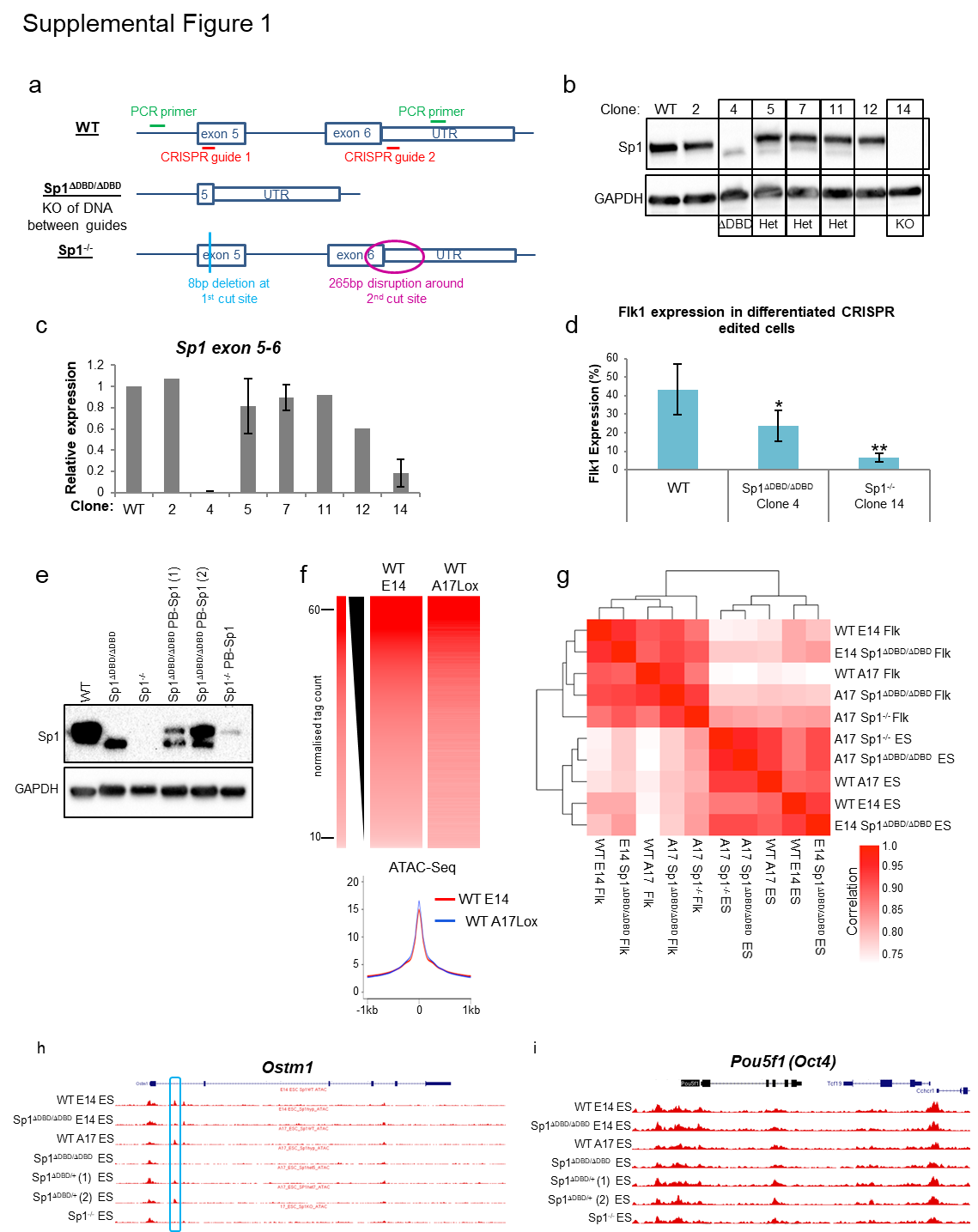
**

**Supplemental Figure 1: Generation of Sp1 mutated ESC lines. Related to Figure 1.**

1. CRISPR strategy for deletion of the Sp1 DBD in A17Lox WT cells and the resulting deletions.
2. Western blot of whole cell extracts from A17Lox WT ESC and CRISPR clones showing levels of Sp1 protein expression and the corresponding GAPDH loading control. Clone numbers are indicated above the blot and the type of deletion indicated below.
3. Sp1 gene expression levels in A17Lox WT cells and the different CRISPR clones using primers directed to exons 5 and 6 of the mouse Sp1 DNA sequence.
4. Flk1 expression in A17Lox WT, Sp1^ΔDBD/ΔDBD^ and Sp1^-/-^ cells as measured by FACS analysis of Flk1-PE staining (n=5, error bars represent standard deviation, * indicates p<0.05, ** indicates p<0.01).
5. Western blot showing rescue of Sp1 protein levels after re-introduction of WT Sp1 protein expression in Sp1^-/-^ and Sp1^ΔDBD/ΔDBD^ CRISPR clones. Sp1^-/-^ and Sp1^ΔDBD/ΔDBD^ ESC were transfected with a PiggyBac vector expressing WT human Sp1 (PB-Sp1).
6. Normalised tag count of accessible chromatin (ATAC) sites in E14 and A17Lox WT ESC lines. Associated average profiles are shown below.
7. Pearson correlation plot of ATAC sites for ESC and Flk1+ cells for WT, Sp1^ΔDBD/ΔDBD^ and Sp1^-/-^ in both E14 and A17Lox ESC backgrounds. Scale indicates the degree of correlation.
8. Screenshot showing the *Ostm1* locus as an example of a region where there is a loss of a hypersensitive site in the Sp1^ΔDBD/ΔDBD^ and Sp1^-/-^ cell lines – indicated by the blue box.
9. Screenshot showing the *Oct4* (*Pou5f1*) locus as an example of a region where hypersensitive sites are similar between the different cell lines.

**
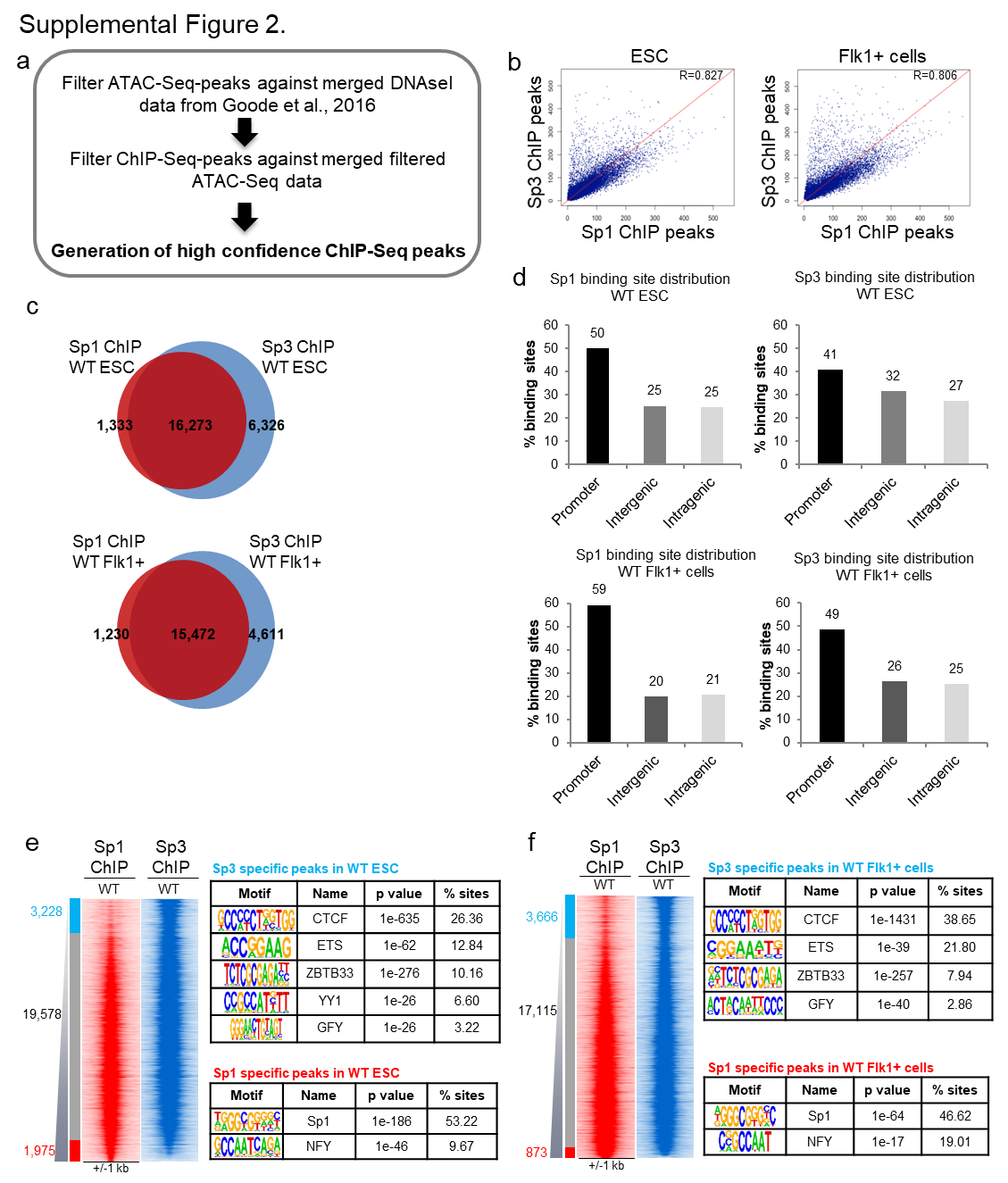
**

**
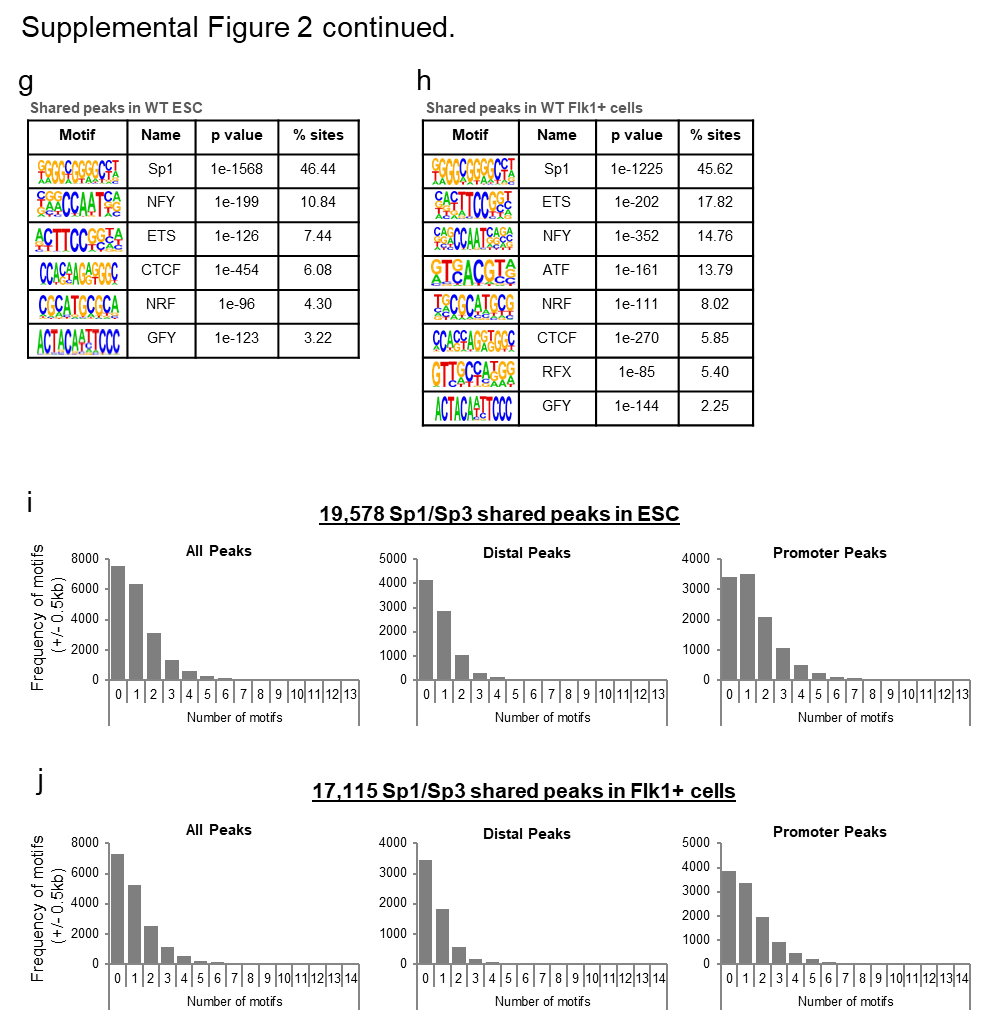
**

**Supplemental Figure 2: The majority of Sp1 and Sp3 binding sites overlap. Related to Figure 2.**

1. Schematic diagram illustrating the identification of high confidence ChIP-seq peaks.
2. Scatter plots of Sp1 vs Sp3 ChIP-seq peaks in A17Lox WT ESC and Flk1+ cells.
3. Venn diagram showing the intersect between Sp1 and Sp3 ChIP-seq peaks in A17Lox WT ESC (top panel) or Flk1+ cells (bottom panel).
4. Bar graphs showing peak distribution for Sp1 and Sp3 ChIP in A17Lox WT ESC and Flk1+ cells.
5. Density plot showing fold change of the peak unions between Sp1 and Sp3 ChIP-seq peaks in A17Lox WT ESC ranked according to the fold change in tag counts. Shown alongside are the highest ranking motifs present in the Sp1 and Sp3 specific peaks.
6. As in e, but for A17Lox WT Flk1+ cells. Shown alongside are the most enriched motifs present in the Sp1 and Sp3 specific peaks.
7. Table showing the most enriched motifs present in the Sp1 and Sp3 shared peaks in A17Lox WT ESC.
8. Table showing the most enriched motifs present in the Sp1 and Sp3 shared peaks in A17Lox WT Flk1+ cells.
9. Bar graphs indicating numbers of motifs within the 19,578 shared peaks in A17Lox WT ESC. Separate graphs show: All peaks, distal peaks and promoter peaks.
10. Bar graphs indicating numbers of motifs within the 17,115 shared peaks in A17Lox WT Flk1+ cells. Separate graphs show: All peaks, distal peaks and promoter peaks.

**
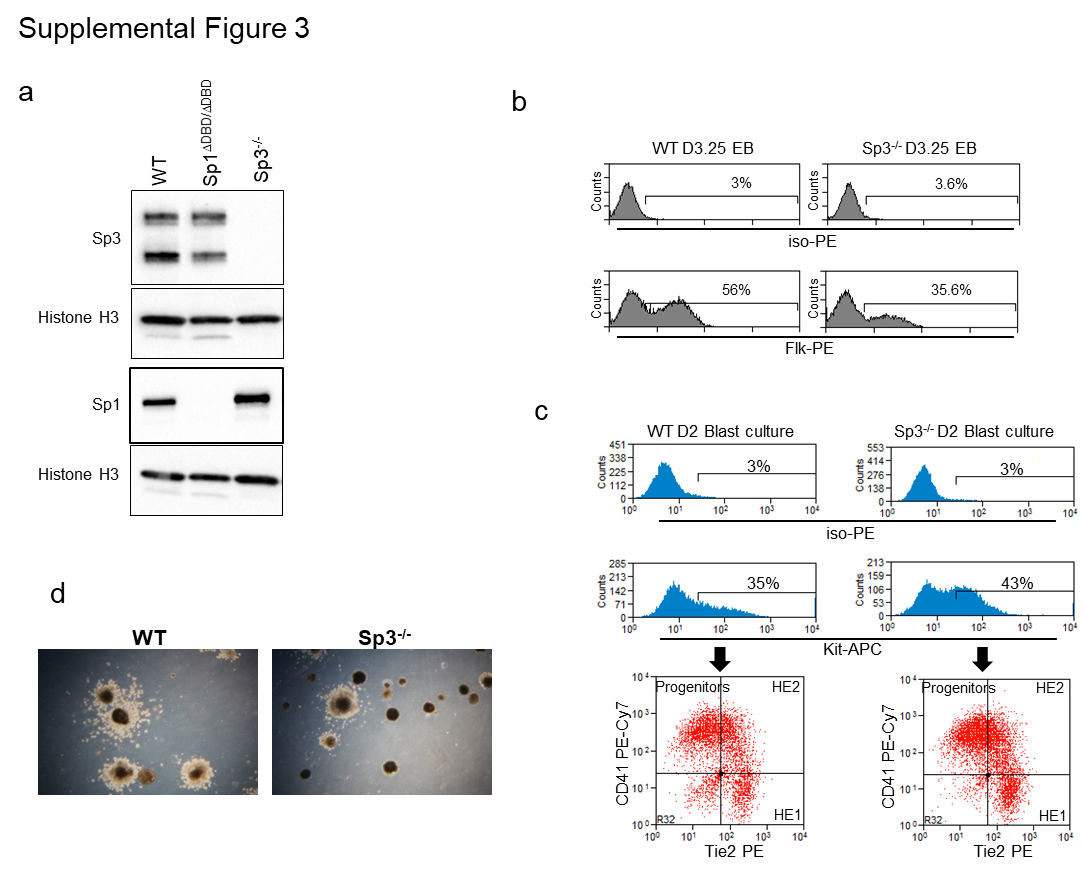
**

**SupplementalFigure 3: Sp3 null ESC also show a myeloid differentiation defect. Related to Figure 3.**

1. Western Blot of nuclear extracts from E14 WT, Sp1^ΔDBD/ΔDBD^ and Sp3^-/-^ ESC showing protein expression of full length Sp1, Sp3 and histone H3 as a loading control.
2. Representative FACS plots showing Flk1-PE staining in E14 WT and Sp3^-/-^ cells at Day 3.25 of EB differentiation.
3. Representative FACS plots showing Kit-APC/Tie2-PE/CD41-PE-Cy7 staining in E14 WT and Sp3^-/-^ Day 2 blast culture cells.
4. Representative images from macrophage release assays from E14 WT and Sp3^-/-^ cells.

**
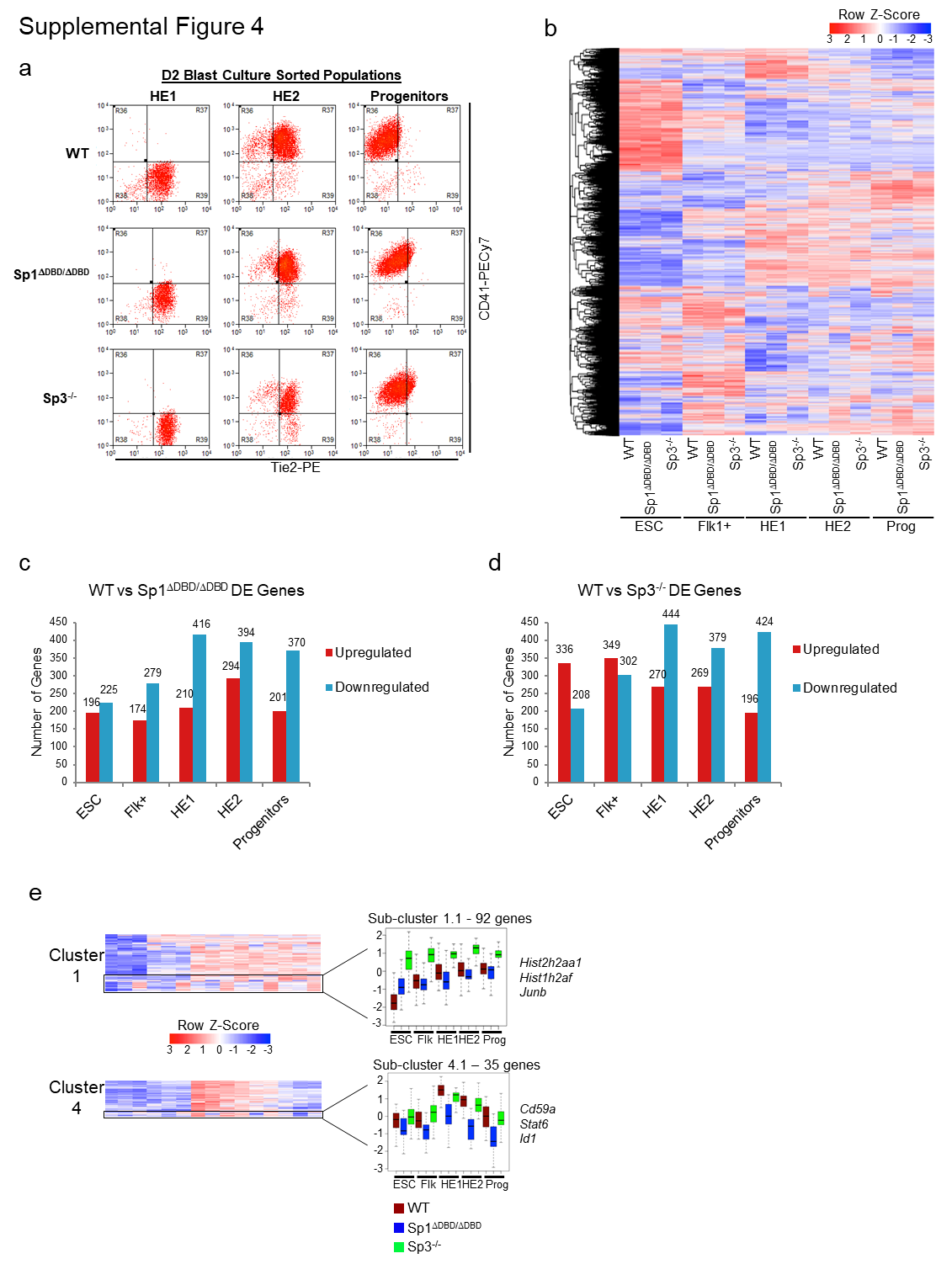
**

**
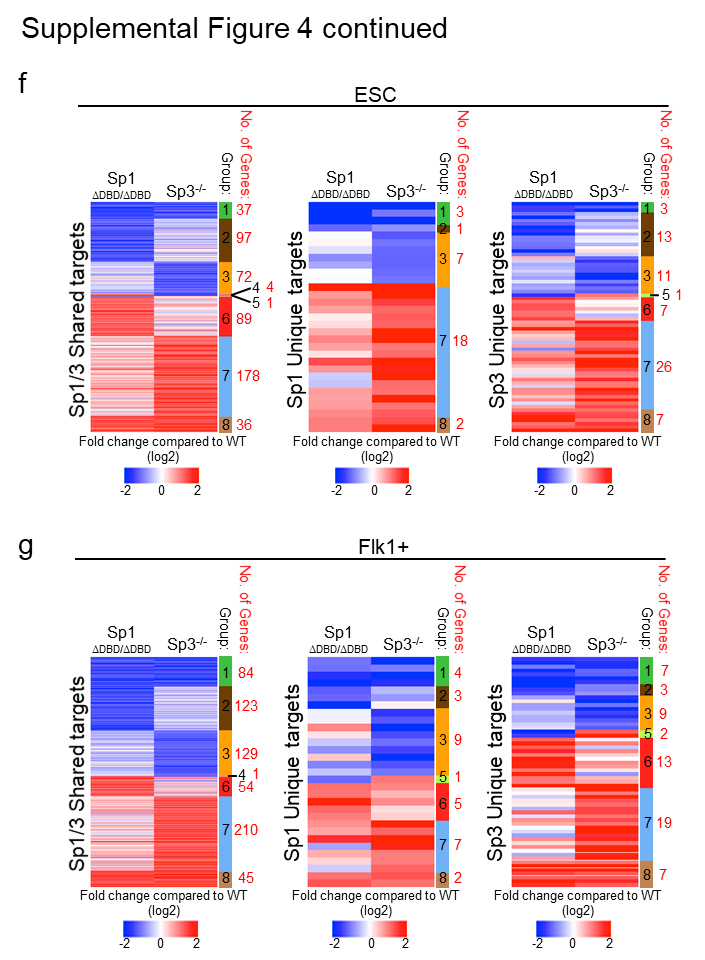
**

**Supplemental Figure 4: Comparison between the deregulation of gene expression in differentiating Sp1^ΔDBD/ΔDBD^ and SP3^-/-^ cells. Related to Figure 4.**

1. Representative FACS plots showing the sorted populations for Day 2 blast culture cells stained with Kit-APC/Tie2-PE/CD41-PE-Cy7 antibodies.
2. Hierarchical clustering of RNA-seq data for all expressed genes for the five populations of E14 WT, Sp1^ΔDBD/ΔDBD^ and Sp3^-/-^ cell lines (ESC, Flk1+, HE1, HE2 and Progenitors).
3. Graph indicating the numbers of up and downregulated genes in Sp1^ΔDBD/ΔDBD^ cell populations compared to E14 WT cells.
4. Graph indicating the numbers of up and downregulated genes in Sp3^-/-^ cell populations compared to E14 WT cells.
5. Highlighted sub-clusters relating to Fig 4A. Left panel shows the relevant cluster and highlighted sub-cluster surrounded by a black box. Right panel shows a box plot representing the gene expression for the indicated sub-cluster. Top panel represents cluster 1: sub-cluster 1.1, bottom panel represents cluster 4: sub-cluster 4.1. Example genes are shown to the right of the box plot.
6. Grouping analysis of differentially regulated genes in ESC that are Sp1/Sp3 targets. Left panel shows Sp1/Sp3 shared target genes. Middle panel shows Sp1 unique targets. Right panel shows Sp3 unique targets. The coloured sidebar indicates the clusters present. The number of genes within each cluster is shown in red beside the sidebar.
7. Grouping analysis of differentially regulated genes in Flk1+ cells that are Sp1/Sp3 targets. Left panel shows Sp1/Sp3 shared target genes. Middle panel shows Sp1 unique targets. Right panel shows Sp3 unique targets. The coloured sidebar indicates the clusters present. The number of genes within each cluster is shown in red beside the sidebar.


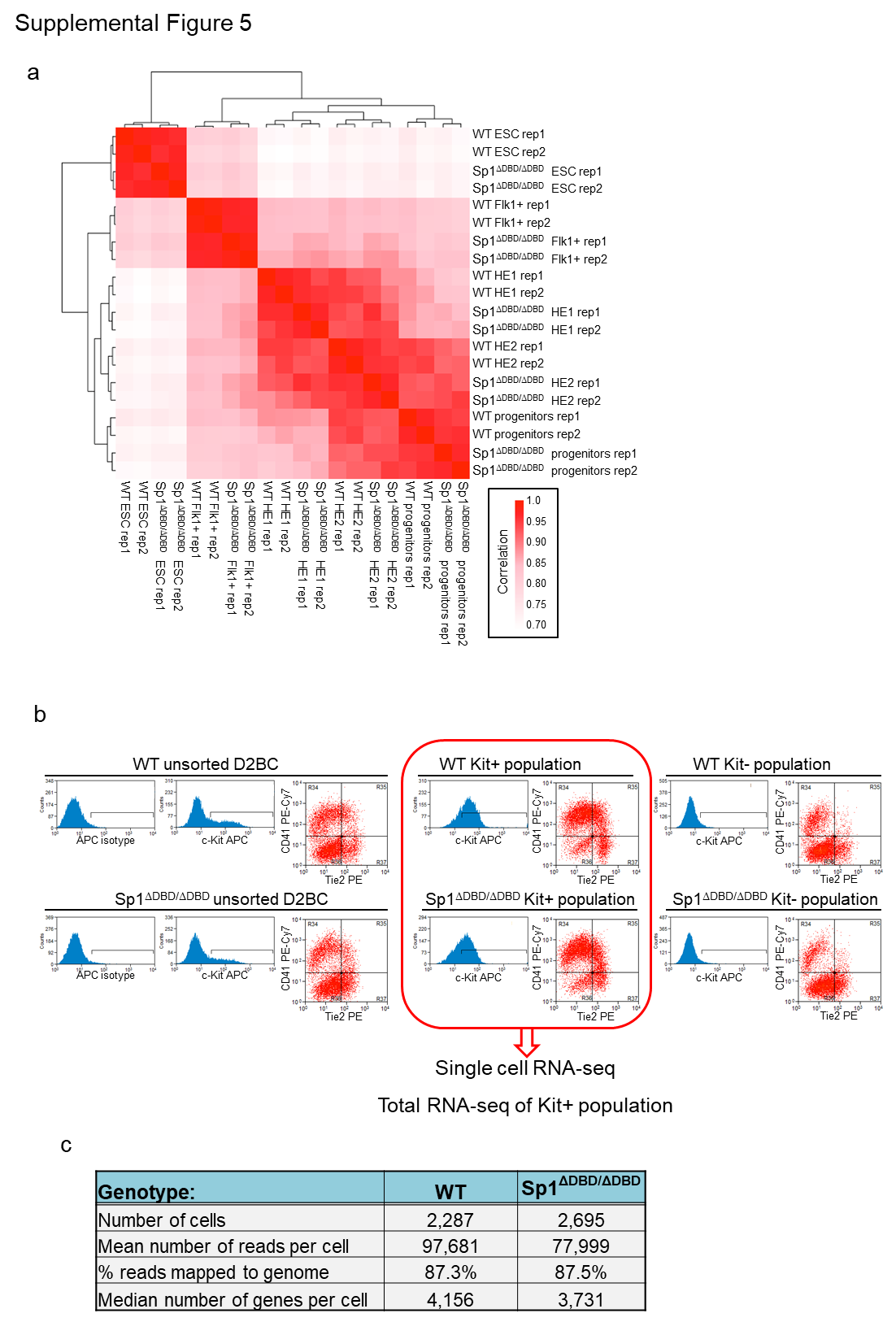


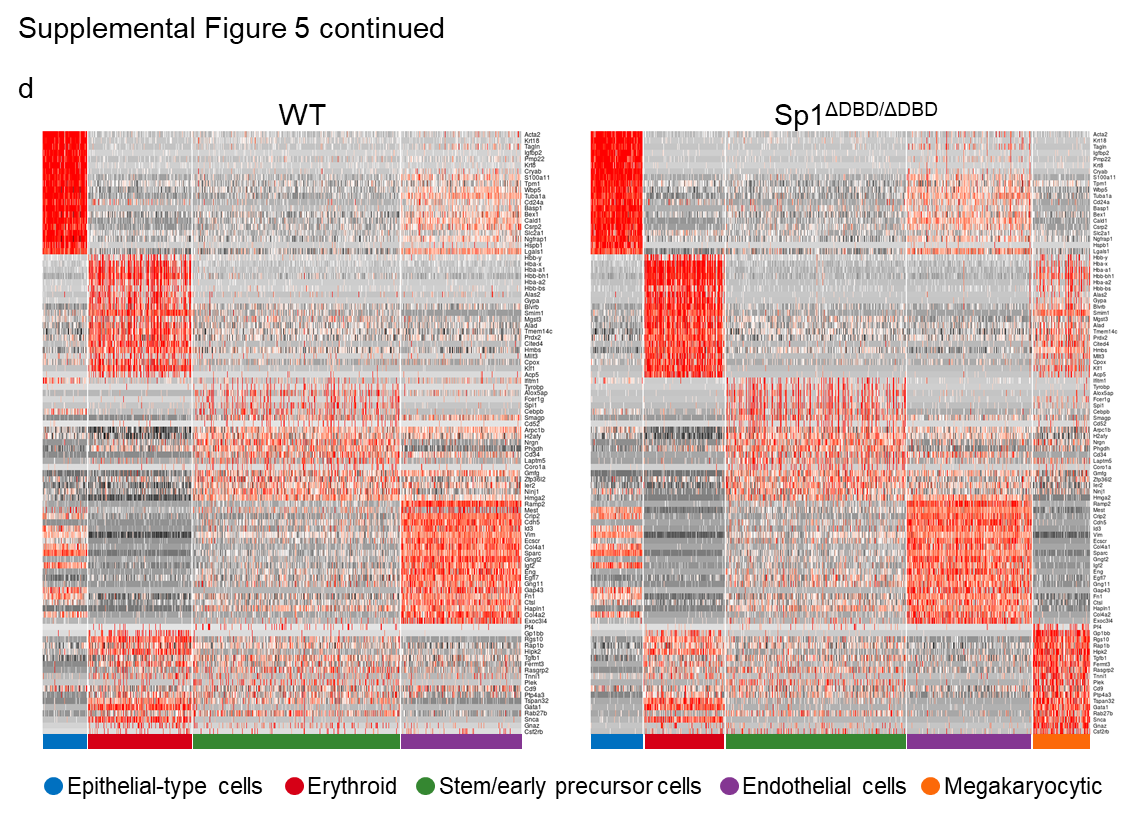


**Supplemental Figure 5: Analysis of gene expression patterns in differentiating Sp1^ΔDBD/ΔDBD^ cells by single cell RNA-seq. Related to Figure 5.**

1. Pearson correlation of RNA-seq data for the five populations of E14 WT and Sp1^ΔDBD/ΔDBD^ cell lines (ESC, Flk1+, HE1, HE2 and Progenitors).
2. Representative FACS plots showing the cell purification strategy of Kit+ sorted cells used for single cell RNA-seq.
3. Table showing sequencing statistics for the Chromium single cell RNA-seq for E14 WT and Sp1^ΔDBD/ΔDBD^.
4. Hierarchical clustering of gene expression from single cell RNA-seq data. The heatmaps show differentially expressed marker genes listed on the right for the different clusters as determined in Fig 5A. Left panel shows E14 WT cells, right panel shows Sp1^ΔDBD/ΔDBD^. The colour code for the cell cluster identity is shown below the heatmaps.


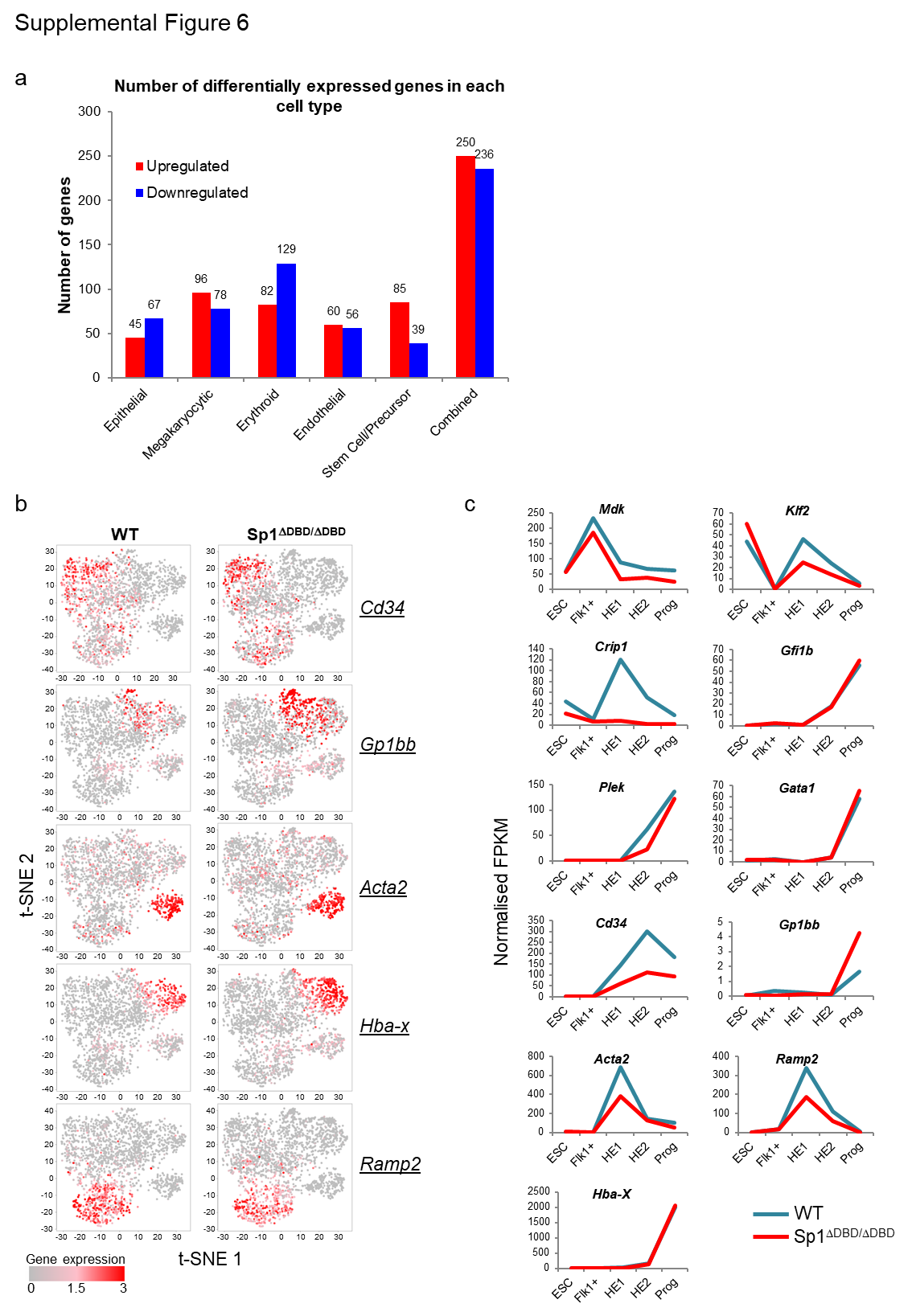


**Supplemental Figure 6: Cluster-specific differential gene expression in differentiating Sp1^ΔDBD/ΔDBD^ cells as measured by single cell RNA-Seq. Related to Figure 6.**

a. Graph depicting the number of differentially expressed genes in Sp1^ΔDBD/ΔDBD^ cells relative to E14 WT cells. Upregulated (red) and downregulated (blue) genes are shown for each cluster and for the combined total.

b. t-distributed stochastic neighbor embedding (t-SNE) visualisation of combined E14 WT and Sp1^ΔDBD/ΔDBD^ populations with each dot representing a single cell. Dots are coloured according to the gene expression values for selected genes as indicated.

c. Normalised FPKM values for selected genes from total RNA-seq performed on the five cell populations as shown in Fig. 4.


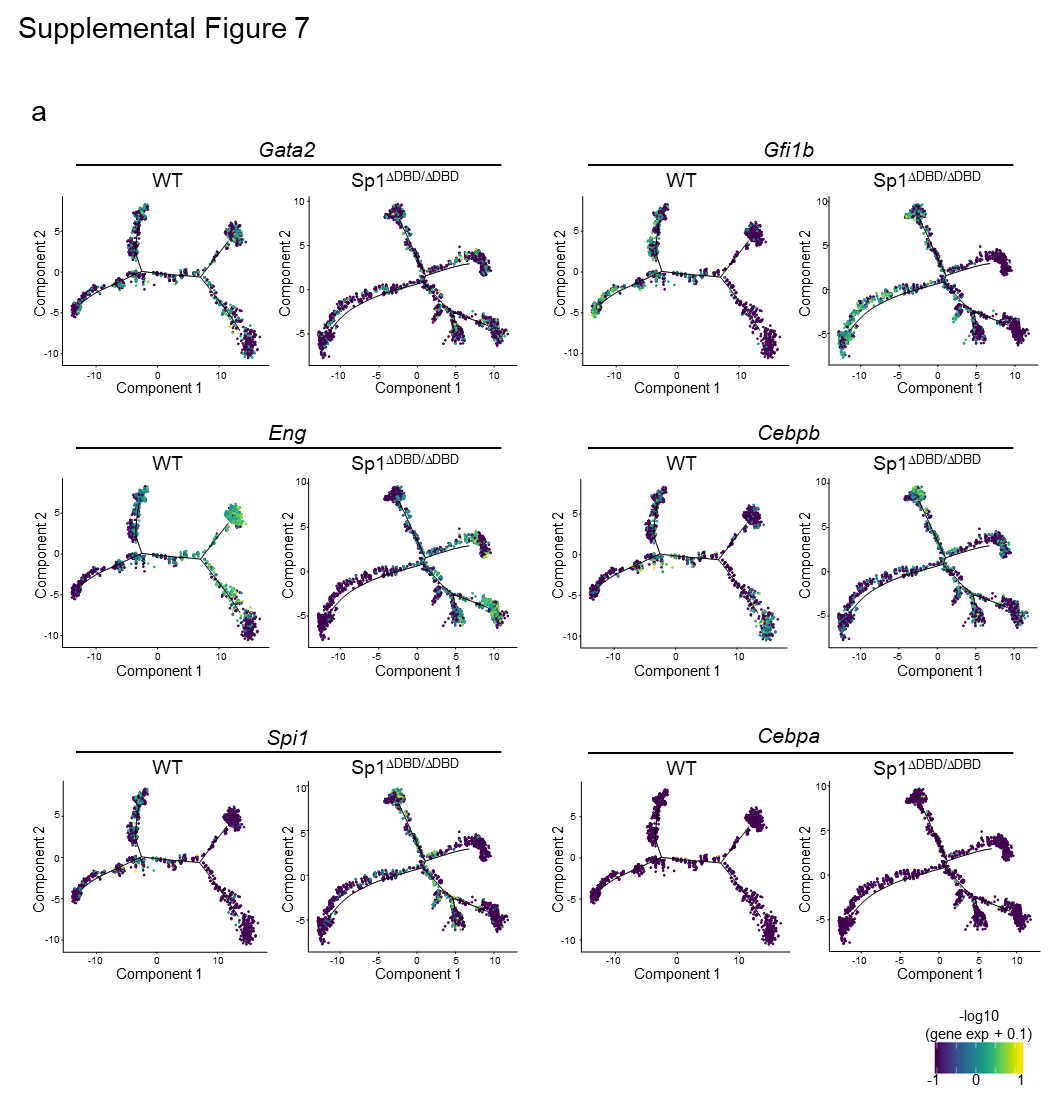


**Supplemental Figure 7: Sp1^ΔDBD/ΔDBD^** **cells show distorted differentiation trajectories. Related to Figure 7.**

1. Expression patterns of selected TFs overlaid on the trajectory plots for E14 WT and Sp1^ΔDBD/ΔDBD^ Day 2 blast culture cell populations. Trajectory plots relate to those shown in Fig. 7a.

**Supplemental Experimental Procedures**

**In vitro differentiation of mouse embryonic stem cells (ESC)**

In vitro differentiation of ESC was performed essentially as described previously (Gilmour et al., 2014; Lancrin et al., 2010; Obier et al., 2016; Regha et al., 2015). Prior to differentiation, the ESC were cultured on gelatin in the absence of feeder cells for two passages. Cells were then differentiated as embryoid bodies (EB) in 15 cm bacterial-grade dishes (Sterilin) at a concentration of 1.25 x10^6^ cells per 50 ml media for between 3 and 3.75 days (depending on the cell line) in Iscove’s Modified Dulbecco’s Medium (IMDM) differentiation medium without Leukemia Inhibitory Factor (LIF) (IMDM supplemented with 15% Foetal Calf Serum (FCS), 100 units/ml Penicillin and 100 μg/ml Streptomycin, 1 mM glutamine, 0.15 mM MTG, 0.18 mg/ml human transferrin (R&D Systems) and 50 μg/ml Ascorbic Acid. TrypLE Express (Gibco, Thermo Fisher) was used to disperse EB to single cells and Flk1+ cells were subsequently purified by magnetic cell sorting using a biotin-conjugated anti-Flk1 antibody (ebioscience), anti-biotin microbeads (Miltenyi Biotec) and MACS columns (Miltenyi Biotec). Purified Flk1+ cells were cultured in gelatinized T150 flasks at 1.6–3 x 10^6^ cells per flask in blast culture medium (IMDM supplemented with 10% FCS, 20% D4T conditioned media, 100 units per ml penicillin and 100 μg per ml streptomycin, 1 mM glutamine, 0.45 mM 1-thioglycerol (MTG), 25 μg per ml ascorbic acid, 0.18 mg per ml human transferrin, 5 ng per ml murine Vascular Endothelial Growth Factor [mVEGF] [Peprotech], 10 ng per ml murine Interleukin 6 [mIL-6] [Peprotech]) for 2 days.

**Primitive Erythrocyte (EryP) assay**

EryP assays were modified from Sturgeon et al., and performed essentially as described previously (Gilmour et al., 2014; Sturgeon et al., 2012). ESC were trypsinised and seeded into base methylcellulose (Stem Cell Technologies M3134) supplemented with 10% FCS, 100 units/ml Penicillin and 100 µg/ml Streptomycin, 1 mM glutamine, 0.15 mM MTG, 10 µg/ml insulin (Sigma), 5% Interleukin 3 (IL-3) conditioned media, 10% Macrophage Colony Stimulating Factor (M-CSF) conditioned media, 100 units/ml Interleukin 1 (IL-1) (Peprotech) to allow formation of EB. EB were harvested and dispersed after 5–10 days and seeded into base methylcellulose supplemented with 10% Plasma Derived Serum (First Link), 5% PFHM (Invitrogen), 100 units per ml Penicillin and 100 μg per ml Streptomycin, 2 mM glutamine, 0.18mg per ml Transferrin, 50 μg per ml ascorbic acid, 0.45 mM MTG and 2 units per ml Erythropoietin (R&D Systems) at 5x10^4^ cells/ml in duplicate 3 cm bacteriological grade dishes. Ery-P colonies were counted on Day 5 after plating.

**qPCR validation of CRISPR clones**

RNA was isolated as for the RNA-seq libraries and cDNA was synthesised using Superscript II and Oligo dT (Thermo Fisher) according to the manufacturer’s instructions. qRT-PCR was performed using SYBR Green PCR Master Mix (Thermo Fisher) and PCR primers directed against exons 5 and 6 of the mouse Sp1 sequence or GAPDH as a normalisation control.

Sp1 exon 5/6 Forward primer: 5’- TCATATTGTGGGAAGCGCTTT

Sp1 exon 5/6 Reverse primer: 5’- CAGGGCAGGCAAATTTCTTCT

GAPDH Forward primer: 5’- ACCTGCCAAGTATGATGACATCA

GAPDH Reverse primer: 5’- GGTCCTCAGTGTAGCCCAAGAT

**Assay for Transposase Accessible Chromatin using sequencing (ATAC-seq) and library generation**

ATAC-seq was performed in ESC and Flk1+ cells essentially as described in Corces et al. 2016 (Corces et al., 2016). Briefly, cells were harvested and 50,000 cells pelleted prior to resuspension in 50 µl ATAC transposition reaction mix containing 2x Tagment DNA Buffer (Illumina), Tn5 transposase (Illumina) and 0.5 µl 1% Digitonin (Promega). DNA was purified using a Minelute Reaction Clean up kit (Qiagen). DNA was PCR amplified using Nextera custom primers and saturation of the PCR reaction avoided by monitoring the reaction to prevent overamplification as described in Buenrostro et al. (Buenrostro et al., 2013)^,^(Buenrostro et al., 2015). Amplified DNA was subsequently purified using Minelute columns or Ampure Beads (Beckman Coulter) prior to validation. Libraries were sequenced in a pool of 12 indexed libraries using a NextSeq 500/550 High Output Kit v2 (150 cycles) for paired end sequencing (Illumina) at the Genomics Birmingham sequencing facility.

**Chromatin Immunoprecipitation (ChIP) and ChIP-seq**

ChIP was performed as described previously (Gilmour et al., 2014; Obier et al., 2016; Regha et al., 2015). For double crosslinking, cells were harvested and washed with PBS prior to a 2-step crosslinking procedure. Cells were incubated for 45 min at room temperature in PBS supplemented with 0.83 mg/ml Di(N-succinimidyl) glutarate (DSG, Sigma). Cells were then washed 4 times with PBS, prior to formaldehyde crosslinking of proteins and DNA for 10 min at RT using 1% formaldehyde (Pierce) in IMDM with 10% FCS. Formaldehyde was quenched by adding 1/10^th^ volume 2 M glycine and crosslinked cells were washed twice in ice-cold PBS. Nuclei were prepared as described in Lefevre et al., 2003(Lefevre et al., 2003), sonicated using a Bioruptor water bath in immunoprecipitation buffer I (25 mM Tris 1 M pH 8.0, 150 mM NaCl, 2 mM EDTA pH 8.0, 1% TritonX-100 and 0.25% SDS). After centrifugation the sheared 0.5–2 kb chromatin fragments (2 x 10^6^ cells) were diluted with 2 volumes immunoprecipitation buffer II (25 mM Tris pH 8.0, 150mM NaCl, 2 mM EDTA pH 8.0, 1% TritonX-100, 7.5% glycerol). Immunoprecipitation was carried out for 2–4 hours at 4°C using 2 μg antibody coupled to 15 μl Protein-G dynabeads (Invitrogen). Following immunoprecipitation, the beads were washed with low salt, high salt, LiCl and TE/NaCl buffers and crosslinks were reversed overnight. DNA was extracted using Ampure beads (Beckman Coulter) and qPCRs were performed to validate ChIP quality. Antibodies used for ChIP: Sp1, Millipore 17-601; Sp3, Santa Cruz sc644X.

Public ChIP-seq and DNase I Hypersensitive Site (DHS) datasets used are described in Supplemental Table 1 below.

**Supplemental Table 1.**

| **ChIP** | **Accession number** | **Author/Ref** |
| --- | --- | --- |
| YY1 | GSE31786 | Vella et al., 2012 (Vella et al., 2012) |
| NFY | GSE56840 | Oldfield et al., 2014 (Oldfield et al., 2014) |
| CTCF | GSM2418860 | Hansen et al., 2017 (Hansen et al., 2017) |
| Esrrb | GSM288355 | Chen et al., 2008 (Chen et al., 2008) |
| Nanog | GSM288345 | Chen et al., 2008 (Chen et al., 2008) |
| Pou5f1 | GSM288346 | Chen et al., 2008 (Chen et al., 2008) |
| Sox2 | GSM288347 | Chen et al., 2008 (Chen et al., 2008) |
| H3K9ac | GSM1000123 | Encode Consortium (Stamatoyannopoulos et al., 2012) |
| H3K27me3 | GSM1000089 | Encode Consortium (Stamatoyannopoulos et al., 2012) |
| H3K4me3 | GSM1003756 | Encode Consortium (Stamatoyannopoulos et al., 2012) |
| H3K27ac | GSM1000126 | Encode Consortium (Stamatoyannopoulos et al., 2012) |
| **DHS** | **Accession number** | **Author/Ref** |
| ESC DHS | GSM1014159 | Encode Consortium (Stamatoyannopoulos et al., 2012) |
| HB DHS | GSM1692782 | Goode et al., 2016 (Goode et al., 2016) |
| HE DHS | GSM1692783 | Goode et al., 2016 (Goode et al., 2016) |
| HP DHS | GSM1692784 | Goode et al., 2016 (Goode et al., 2016) |

**Bioinformatic Analysis**

**ATAC-Seq data processing**

Raw sequencing reads were assessed for sequencing quality using FastQC (available from https://www.bioinformatics.babraham.ac.uk/projects/fastqc/). Nextera sequencing adapters were then removed with Cutadapt version 1.13 (Martin, 2011). Sequences were aligned to the mouse genome (mm10) with Bowtie version 2.3.1 (Langmead and Salzberg, 2012) with the parameter --very-sensitive-local. Only reads that could be uniquely aligned to the genome were retained for further analysis. Further information on numbers of reads, peaks and alignment can be found in Supplemental Data-set 4.

Open chromatin regions (peaks) were identified using MACS 1.4.2 (Zhang et al., 2008), with the options ‑‑keep‑dup=all -w -S. These peaks were then compared against a previously generated DNaseI dataset consisting of open chromatin regions across various stages of hematopoietic differentiation (Goode et al., 2016). Only ATAC peaks that could be aligned to these DNaseI hypersensitive sites were retained for further analysis. Peaks from ATAC-seq replicates were combined using the merge function in Bedtools (Quinlan and Hall, 2010) and were used in all further analyses.

**Chip-Seq data processing**

Sequencing reads from ChIP-Seq experiments were processed and aligned to the genome in the same way as the ATAC-Seq data, with the exception of the peak calling step in which MACS was used with the parameter --keep-dup=auto. Only ChIP-Seq peaks that were found within open chromatin, as determined by the ATAC-Seq data, were retained for further analysis. Further information on numbers of reads, peaks and alignment can be found in Supplemental Data-set 4.

**ATAC-Seq and ChIP-Seq data analysis**

Peaks from both ATAC and ChIP-seq experiments were annotated to their closest gene using the annotatePeaks.pl function in the homer software package version 4.9.1 (Heinz et al., 2010). Peaks were classified as within the promoter if they were within 2 kb of the transcription start site (TSS), and as distal peaks otherwise. A de-novo motif search was carried out within these peaks using the findMotifsGenome.pl function in homer. Only motifs that had a p-value <0.01, and that were found in more than 2% of sites were retained for further analysis. The chromosomal coordinates of motifs that occurred within ChIP-Seq peaks were determined using the annotatePeaks.pl function in homer, using the -m -mbed options. The distances between pairs of motifs were measured using the closest function in bedtools, using the centre position of each motif to represent its chromosomal position.

ATAC peak unions were constructed by merging peaks that had summit positions within 200 bp of each other. In these cases a new open chromatin region was defined with a summit position at the mid-point between the summits of the original peaks. These positions were then used in all further analyses.

To identify differentially accessible ATAC peaks, a peak union was first created between the pair of samples being considered in the analysis. The average read density in these peaks was then retrieved from the wiggle files created by MACS using the annotatePeaks.pl function in homer. Read counts were normalized by total read count in R version 3.5.1. A chromatin region was deemed to be differentially accessible if the fold-difference between the normalized read counts was >2 fold. To create density plots, peaks were first ordered according to fold-difference and the read density in a 2 kb region centred on the peak was calculated using the annotatePeaks.pl function in homer, with the options ‑size 2000 -hist 10 ‑ghist. These were then plotted as a heatmap using Java TreeView (Saldanha, 2004).

Hierarchical clustering of ATAC-Seq data was carried out on the union of all peaks across all of the differentiation stages. The average read count across a 400bp window centred on the peaks was retrieved from the wiggle files produced by MACS using the annotatePeaks.pl function in homer. These were then normalized by total read count across all datasets, and further log2-transformed. Pearson correlations were calculated between all pairs of samples, and clustered using complete linkage clustering of the Euclidean distances. The results of this clustering was plotted as a heatmap in R.

**RNA-Seq data analysis**

Sequencing reads from RNA-Seq experiments were assessed for quality using the FastQC software. Sequencing adapters and low quality bases were trimmed from the raw reads using trimmomatic version 0.32 (Bolger et al., 2014). These processed reads were then aligned to the mouse genome (mm10) with Hisat2 version 2.1.0 (Kim et al., 2015) using default parameters. Gene expression was measured as Fragments Per Kilobase per Million mapped reads (FPKM) using stringtie (Pertea et al., 2015) with default parameters. Only genes that had an FPKM value > 1 in at least one sample were retained for further analysis. Raw FPKM values were normalized using quantile normalization using the preprocessCore package in R. These FPKM values were then log2-transformed, with a pseudo-count of 0.1 being added to the FPKM values prior to transformation. Pairwise Pearson correlation values were calculated in R, and hierarchically clustered using complete linkage clustering of the Euclidean distances, and finally plotted as a heatmap.

Differentially expressed genes were identified by comparing either the Sp1^ΔDBD/ΔDBD^ or Sp3^-/-^ against the E14 WT cells at each differentiation stage. A gene was considered to be differentially expressed if it had a fold-change >2. Gene Ontology (GO) term analysis was carried out for each of the differentially expressed gene sets using the ClueGO plugin for Cytoscape (Bindea et al., 2009; Shannon et al., 2003). GO terms with a Benjamini-Hochberg corrected p-value <0.05 was considered to be significantly enriched.

Covariance analysis was conducted only on genes that were found to be differentially expressed between E14 WT and Sp1^ΔDBD/ΔDBD^ or Sp3^-/-^ at any of the differentiation stages. FPKM values were transformed to a Z-scale using the scale function in R, and then hierarchically clustered using complete linkage clustering of the Euclidean distances. Clusters that correspond to groups of genes that show a similar pattern of gene expression across differentiation were then extracted from the dendrogram using the DynamicTreeCut package in R using the hybrid method (Langfelder et al., 2008).

**Single-cell RNA-Seq analysis**

Illumina base call (BCL) files were de-multiplexed and converted to fastq format using the mkfastq function in the CellRanger software from 10x genomics (version 2.1.1). These files were then aligned to the mouse genome (mm10) and transcripts were quantified using the count function in CellRanger. Unique Molecular Identifier (UMI) counts were normalized using Monocle version 2.8.0 in R (Qiu et al., 2017; Trapnell et al., 2014). Only cells that had at least 2000 detectable genes were retained for further analysis. Cells were clustered using t-distributed stochastic neighbor embedding (t-SNE), and cell populations were identified using the clusterCells function in Monocle. Cell trajectory (pseudotime) analysis was carried out by first reducing the dimensions of the data using the Discriminative Dimensionality Reduction with Trees (DDRTree) method, and ordered along a pseudotime trajectory using the orderCells function in Monocle. The resulting trajectories were then plotted using the plot_cell_trajectory function.

To identify cell population marker genes, the CellDataSet object from Monocle was first exported to the Seurat package (version 2.3.4) in R (Butler et al., 2018) using the exportCDS function in Monocle. The FindMarkers function in Seurat was then used to identify marker genes, which correspond to genes that are significantly up-regulated (adj. p-value <0.05) in each cell population compared to the others. From the marker genes identified, the top 20 most highly up-regulated genes were chosen from each cell population and the expression of these genes across all cells were plotted as a heatmap using the DoHeatmap function in Seurat.

Differential gene expression analysis comparing the E14 WT and Sp1^ΔDBD/ΔDBD^ single cell datasets was conducted by first merging the two datasets using the MergeSeurat function in Seurat. Canonical correlation analysis (CCA) was then used to identify common sources of variation between the two datasets (Butler et al., 2018). These CCA subspaces were then aligned using the AlignSubspace function in Seurat, and clustered using t-SNE. Cell clusters were identified using the FindClusters function in Seurat. Importantly, the cell populations found by Seurat showed good correspondence to those that were previously found by Monocle, as shown by the presence of key marker genes in each of these cell populations. Finally, differentially expressed genes were identified in each of the cell populations using the FindMarkers function in Seurat. Genes with an adjusted p-value <0.05 were considered to be significantly differentially expressed.

**Supplemental References**

Bindea, G., Mlecnik, B., Hackl, H., Charoentong, P., Tosolini, M., Kirilovsky, A., Fridman, W.H., Pages, F., Trajanoski, Z., and Galon, J. (2009). ClueGO: a Cytoscape plug-in to decipher functionally grouped gene ontology and pathway annotation networks. Bioinformatics *25*, 1091-1093.

Bolger, A.M., Lohse, M., and Usadel, B. (2014). Trimmomatic: a flexible trimmer for Illumina sequence data. Bioinformatics *30*, 2114-2120.

Buenrostro, J.D., Giresi, P.G., Zaba, L.C., Chang, H.Y., and Greenleaf, W.J. (2013). Transposition of native chromatin for fast and sensitive epigenomic profiling of open chromatin, DNA-binding proteins and nucleosome position. Nat Methods *10*, 1213-1218.

Buenrostro, J.D., Wu, B., Chang, H.Y., and Greenleaf, W.J. (2015). ATAC-seq: A Method for Assaying Chromatin Accessibility Genome-Wide. Curr Protoc Mol Biol *109*, 21 29 21-29.

Butler, A., Hoffman, P., Smibert, P., Papalexi, E., and Satija, R. (2018). Integrating single-cell transcriptomic data across different conditions, technologies, and species. Nat Biotechnol *36*, 411-420.

Chen, X., Xu, H., Yuan, P., Fang, F., Huss, M., Vega, V.B., Wong, E., Orlov, Y.L., Zhang, W., Jiang, J.*, et al.* (2008). Integration of external signaling pathways with the core transcriptional network in embryonic stem cells. Cell *133*, 1106-1117.

Corces, M.R., Buenrostro, J.D., Wu, B., Greenside, P.G., Chan, S.M., Koenig, J.L., Snyder, M.P., Pritchard, J.K., Kundaje, A., Greenleaf, W.J.*, et al.* (2016). Lineage-specific and single-cell chromatin accessibility charts human hematopoiesis and leukemia evolution. Nat Genet *48*, 1193-1203.

Gilmour, J., Assi, S.A., Jaegle, U., Kulu, D., van de Werken, H., Clarke, D., Westhead, D.R., Philipsen, S., and Bonifer, C. (2014). A crucial role for the ubiquitously expressed transcription factor Sp1 at early stages of hematopoietic specification. Development *141*, 2391-2401.

Goode, D.K., Obier, N., Vijayabaskar, M.S., Lie, A.L.M., Lilly, A.J., Hannah, R., Lichtinger, M., Batta, K., Florkowska, M., Patel, R.*, et al.* (2016). Dynamic Gene Regulatory Networks Drive Hematopoietic Specification and Differentiation. Dev Cell *36*, 572-587.

Hansen, A.S., Pustova, I., Cattoglio, C., Tjian, R., and Darzacq, X. (2017). CTCF and cohesin regulate chromatin loop stability with distinct dynamics. Elife *6*.

Heinz, S., Benner, C., Spann, N., Bertolino, E., Lin, Y.C., Laslo, P., Cheng, J.X., Murre, C., Singh, H., and Glass, C.K. (2010). Simple combinations of lineage-determining transcription factors prime cis-regulatory elements required for macrophage and B cell identities. Mol Cell *38*, 576-589.

Kim, D., Langmead, B., and Salzberg, S.L. (2015). HISAT: a fast spliced aligner with low memory requirements. Nat Methods *12*, 357-360.

Lancrin, C., Sroczynska, P., Serrano, A.G., Gandillet, A., Ferreras, C., Kouskoff, V., and Lacaud, G. (2010). Blood cell generation from the hemangioblast. J Mol Med (Berl) *88*, 167-172.

Langfelder, P., Zhang, B., and Horvath, S. (2008). Defining clusters from a hierarchical cluster tree: the Dynamic Tree Cut package for R. Bioinformatics *24*, 719-720.

Langmead, B., and Salzberg, S.L. (2012). Fast gapped-read alignment with Bowtie 2. Nat Methods *9*, 357-359.

Lefevre, P., Melnik, S., Wilson, N., Riggs, A.D., and Bonifer, C. (2003). Developmentally regulated recruitment of transcription factors and chromatin modification activities to chicken lysozyme cis-regulatory elements in vivo. Mol Cell Biol *23*, 4386-4400.

Martin, M. (2011). Cutadapt removes adapter sequences from high-throughputsequencing reads. EMBnet Journal *17*, 10-12.

Obier, N., Cauchy, P., Assi, S.A., Gilmour, J., Lie, A.L.M., Lichtinger, M., Hoogenkamp, M., Noailles, L., Cockerill, P.N., Lacaud, G.*, et al.* (2016). Cooperative binding of AP-1 and TEAD4 modulates the balance between vascular smooth muscle and hemogenic cell fate. Development *143*, 4324-4340.

Oldfield, A.J., Yang, P., Conway, A.E., Cinghu, S., Freudenberg, J.M., Yellaboina, S., and Jothi, R. (2014). Histone-fold domain protein NF-Y promotes chromatin accessibility for cell type-specific master transcription factors. Mol Cell *55*, 708-722.

Pertea, M., Pertea, G.M., Antonescu, C.M., Chang, T.C., Mendell, J.T., and Salzberg, S.L. (2015). StringTie enables improved reconstruction of a transcriptome from RNA-seq reads. Nat Biotechnol *33*, 290-295.

Qiu, X., Mao, Q., Tang, Y., Wang, L., Chawla, R., Pliner, H.A., and Trapnell, C. (2017). Reversed graph embedding resolves complex single-cell trajectories. Nat Methods *14*, 979-982.

Quinlan, A.R., and Hall, I.M. (2010). BEDTools: a flexible suite of utilities for comparing genomic features. Bioinformatics *26*, 841-842.

Regha, K., Assi, S.A., Tsoulaki, O., Gilmour, J., Lacaud, G., and Bonifer, C. (2015). Developmental-stage-dependent transcriptional response to leukaemic oncogene expression. Nat Commun *6*, 7203.

Saldanha, A.J. (2004). Java Treeview--extensible visualization of microarray data. Bioinformatics *20*, 3246-3248.

Shannon, P., Markiel, A., Ozier, O., Baliga, N.S., Wang, J.T., Ramage, D., Amin, N., Schwikowski, B., and Ideker, T. (2003). Cytoscape: a software environment for integrated models of biomolecular interaction networks. Genome Res *13*, 2498-2504.

Stamatoyannopoulos, J.A., Snyder, M., Hardison, R., Ren, B., Gingeras, T., Gilbert, D.M., Groudine, M., Bender, M., Kaul, R., Canfield, T.*, et al.* (2012). An encyclopedia of mouse DNA elements (Mouse ENCODE). Genome Biol *13*, 418.

Sturgeon, C.M., Chicha, L., Ditadi, A., Zhou, Q., McGrath, K.E., Palis, J., Hammond, S.M., Wang, S., Olson, E.N., and Keller, G. (2012). Primitive erythropoiesis is regulated by miR-126 via nonhematopoietic Vcam-1+ cells. Dev Cell *23*, 45-57.

Trapnell, C., Cacchiarelli, D., Grimsby, J., Pokharel, P., Li, S., Morse, M., Lennon, N.J., Livak, K.J., Mikkelsen, T.S., and Rinn, J.L. (2014). The dynamics and regulators of cell fate decisions are revealed by pseudotemporal ordering of single cells. Nat Biotechnol *32*, 381-386.

Vella, P., Barozzi, I., Cuomo, A., Bonaldi, T., and Pasini, D. (2012). Yin Yang 1 extends the Myc-related transcription factors network in embryonic stem cells. Nucleic Acids Res *40*, 3403-3418.

Zhang, Y., Liu, T., Meyer, C.A., Eeckhoute, J., Johnson, D.S., Bernstein, B.E., Nusbaum, C., Myers, R.M., Brown, M., Li, W.*, et al.* (2008). Model-based analysis of ChIP-Seq (MACS). Genome Biol *9*, R137.
