# Supplementary material for: Robust hematopoietic specification requires the ubiquitous Sp1 and Sp3 transcription factors
Source: Epigenetics Chromatin. 2019 Jun 4;12:33. doi: 10.1186/s13072-019-0282-9 (PMC6547542; doi:10.1186/s13072-019-0282-9)
Supplement: Supplementary file 5 — Additional file 5. Sequencing data list [file 13072_2019_282_MOESM5_ESM.pdf]

Supplementary Dataset 4: Sequencing data

ESC-ATAC-Seq	No. Reads	mapped reads	alignment %	peaks	DHS filtered peaks
**Merged filtered peaks**					37834
A17Lox WT	119220592	116246038	97.51	36603	24671
E14 WT	241328968	233172049	96.62	52187	32017
A17Lox Sp1$^{+/\Delta DBD}$ (1)	126086922	123224749	97.73	43303	27953
A17Lox Sp1$^{+/\Delta DBD}$ (2)	120502300	117170411	97.24	53672	32025
A17Lox Sp1$^{-/-}$	142343688	138464823	97.28	40998	26507
A17Lox Sp1$^{\Delta DBD/\Delta DBD}$	110603648	103016238	93.14	36966	24437
E14 Sp1$^{\Delta DBD/\Delta DBD}$	197196262	190767664	96.74	50030	30522

Sp1 ChIP data in ESC	No. Reads	mapped reads	alignment %	peaks	high confidence peaks
A17Lox WT	22763937	20588794	90.44	33021	17606
A17Lox Sp1$^{+/\Delta DBD}$ (1)	29910896	27026859	90.36	42283	17652
A17Lox Sp1$^{+/\Delta DBD}$ (2)	33050437	29069050	87.95	23848	15654
A17Lox Sp1$^{\Delta DBD/\Delta DBD}$					

Sp3 ChIP data in ESC	No. Reads	mapped reads	alignment %	peaks	high confidence peaks
A17Lox WT	22332095	21634384	96.88	37652	22381
A17Lox Sp3 ChIP in Sp1$^{\Delta DBD/\Delta DBD}$	27599919	26695646	96.72	31257	19519
A17Lox Sp3 ChIP in Sp1$^{+/\Delta DBD}$ (1)	23478817	22839127	97.28	41100	23526
A17Lox Sp3 ChIP in Sp1$^{+/\Delta DBD}$ (2)	28238633	27542242	97.53	31614	20438
A17Lox Sp3 ChIP in Sp1$^{-/-}$	32364500	31712735	97.99	41052	23224

Flk1 ATAC-Seq	No. Reads	mapped reads	alignment %	peaks	DHS filtered peaks
**Merged filtered peaks**					32759
A17Lox WT	89251922	86712705	97.16	38324	30188
A17Lox Sp1$^{+/\Delta DBD}$ 1	113505040	110820646	97.64	33763	27487
A17Lox Sp1$^{+/\Delta DBD}$ 2	86405570	83994855	97.21	28902	24424
A17Lox Sp1$^{\Delta DBD/\Delta DBD}$	95677704	93089622	97.3	31808	26249
A17Lox Sp1$^{-/-}$	115788284	106884165	92.31	22587	18530
E14 WT	82811374	80062036	96.68	37504	31120
E14 Sp1$^{\Delta DBD/\Delta DBD}$	82732522	79944436	96.63	32234	27042

Sp1 ChIP data in Flk1 cells	No. Reads	mapped reads	alignment %	peaks	high confidence peaks
A17Lox WT Sp1 ChIP	31967487	30474010	95.33	20055	16702
A17Lox Sp1$^{+/\Delta DBD}$ (1) Sp1 ChIP	36766368	35422322	96.34	9688	9252
A17Lox Sp1$^{+/\Delta DBD}$ (2) Sp1 ChIP	39894821	38378452	96.2	8851	8485

Sp3 ChIP data in Flk1 cells	No. Reads	mapped reads	alignment %	peaks	high confidence peaks
A17Lox WT Sp3 ChIP	41922325	39713976	94.73	31505	20053
A17Lox Sp1$^{\Delta DBD/\Delta DBD}$ Sp3 ChIP	31759643	30975632	97.53	22132	17288
A17Lox Sp1$^{+/\Delta DBD}$ (1) Sp3 ChIP	41492766	39874282	96.1	36877	21541
A17Lox Sp1$^{+/\Delta DBD}$ (2) Sp3 ChIP	44082985	42606458	96.65	28461	19477
